# Microbial and mineral interactions decouple litter quality from soil organic matter formation

**DOI:** 10.1038/s41467-024-54446-0

**Published:** 2024-11-20

**Authors:** Dafydd M. O. Elias, Kelly E. Mason, Tim Goodall, Ashley Taylor, Pengzhi Zhao, Alba Otero-Fariña, Hongmei Chen, Caroline L. Peacock, Nicholas J. Ostle, Robert Griffiths, Pippa J. Chapman, Joseph Holden, Steve Banwart, Niall P. McNamara, Jeanette Whitaker

**Affiliations:** 1 UK Centre for Ecology & Hydrology, Lancaster Environment Centre, Library Avenue, Bailrigg, Lancaster LA1 4AP UK; 2 UK Centre for Ecology & Hydrology, MacLean Building, Benson Lane, Crowmarsh Gifford, Wallingford OX10 8BB UK; 3 School of Earth and Environment, University of Leeds, Leeds, LS2 9JT UK; 4 Lancaster Environment Centre, Lancaster University, Library Ave, Bailrigg, Lancaster LA1 4YQ UK; 5 School of Environmental and Natural Sciences, Bangor University, Bangor, Gwynedd LL57 2DG UK; 6 water@leeds, School of Geography, University of Leeds, Leeds, LS2 9JT UK; 7 Global Food and Environment Institute, University of Leeds, Leeds, LS2 9JT UK; 8 Present Address: Earth and Life Institute, Université Catholique de Louvain, 1348 Louvain-la-Neuve, Belgium; 9 Present Address: CRETUS, University of Santiago de Compostela, 15782 Santiago de Compostela, Spain

**Keywords:** Carbon cycle, Geochemistry

## Abstract

Current understanding of soil carbon dynamics suggests that plant litter quality and soil mineralogy control the formation of mineral-associated soil organic carbon (SOC). Due to more efficient microbial anabolism, high-quality litter may produce more microbial residues for stabilisation on mineral surfaces. To test these fundamental concepts, we manipulate soil mineralogy using pristine minerals, characterise microbial communities and use stable isotopes to measure decomposition of low- and high-quality litter and mineral stabilisation of litter-C. We find that high-quality litter leads to less (not more) efficient formation of mineral-associated SOC due to soil microbial community shifts which lower carbon use efficiency. Low-quality litter enhances loss of pre-existing SOC resulting in no effect of litter quality on total mineral-associated SOC. However, mineral-associated SOC formation is primarily controlled by soil mineralogy. These findings refute the hypothesis that high-quality plant litters form mineral-associated SOC most efficiently and advance our understanding of how mineralogy and litter-microbial interactions regulate SOC formation.

## Abstract (web summary)

This study challenges the hypothesis that high-quality plant litters form stable, mineral-associated soil organic carbon most efficiently, providing evidence that litter-microbial interactions and soil mineralogy regulate soil organic carbon formation.

## Introduction

Soil organic matter (SOM) is the largest terrestrial carbon (C) pool and is predominantly formed from the continuous input of above and belowground plant C to soils^1^. Most plant-derived C is mineralized and respired by soil microorganisms over short timescales (days to years). However, a proportion is stabilised and remains in soils for much longer (centuries to millennia)^2^. The mechanisms to explain why some SOM persists in soils while most is more rapidly mineralized are complex and still largely unresolved^3^. Land-use change and land management have resulted in the historical loss of 40–90 Pg soil organic C (SOC) globally^4,5^. Therefore, elucidating mechanisms that control SOM persistence is crucial to inform land management options that could increase SOC stocks for climate change mitigation^6,7^ and address uncertainties in soil C feedbacks to climate change^8^.

SOM consists of a complex mixture of fragmented plant tissues and microbial residues within the mineral soil matrix^9^. This mixture consists of molecules varying in size, degradation extent, microbial accessibility, and residence time. Mineral-associated organic matter (MAOM) forms from plant or microbial-derived low molecular weight compounds and comprises the largest reservoir of organic C in mineral soils globally^10,11^. MAOM is considered relatively persistent due to physical (occlusion within micropores and aggregates) and chemical (presence and chemistry of mineral surfaces) protective mechanisms that decrease microbial decomposer access to organic molecules. However, not all MAOM persists in soils over long timescales as root exudates can mobilize MAOM^12,13^ and changes to the soil environment (e.g. redox, pH) can promote desorption by changing the solubility and surface charge of minerals^14^. Particulate organic matter (POM) comprises primarily lightweight fragments of plant material that generally turnover faster than MAOM although this varies across ecosystems^10^. POM can also turnover more slowly and contribute to persistent soil C through occlusion of litter fragments within soil aggregates^15–17^. Chemically, POM and MAOM are quite distinct and are proposed as useful operational definitions to facilitate the study of soil C dynamics^18^.

Litter chemistry, in particular the C:N ratio, is a primary control on litter decomposition rates^19^. Low-quality litter (defined as litter with low mineralization rates) promotes POM formation^15,16^ and was previously thought to control SOM persistence due to molecular characteristics that limit decomposition^20–22^. However, high-quality litter (defined as litter with high mineralization rates) is now thought to promote MAOM formation and persistence, by maximising the synthesis of microbial products and residues (often referred to as necromass) that can form a substantial proportion of MAOM^23–25^. Cotrufo, Wallenstein^26^ proposed the Microbial Efficiency-Matrix Stabilization (MEMS) framework to integrate plant litter decomposition and SOM stabilisation. This framework proposed that interactions between SOM and the mineral matrix act as the primary control on organic matter stabilisation over longer timescales but a microbial filter or microbial carbon pump sensu Liang, Schimel^27^ controls the flow of C and nitrogen (N) from plant litter to SOM through the effects of substrate quality on microbial carbon use efficiency (CUE). Microbial CUE describes the ratio between C allocated to biosynthesis and total C assimilated and tends to decline as soil nutrient availability decreases^28,29^. Thus the MEMS framework proposes that MAOM should be formed more efficiently (more MAOM formed, and less CO_2_ produced per unit of litter C) in high-quality litter environments compared to low-quality litter environments. However, this is not supported by empirical evidence as reviews of controlled experiments suggest that the effect of litter quality on SOM stabilisation is inconsistent, and that high quality litter is not always stabilised more efficiently by the mineral soil matrix relative to low quality litter^30–33^. Moreover, the litter quality effect varies across studies conducted under different environmental conditions and different soil types^31,34–41^. To resolve uncertainties regarding the relationship between litter quality and SOM persistence requires greater consideration of the interactions between litter quality, microbes, the mineral matrix, and the abiotic environment.

Litter-microbial interactions are one mechanism proposed to explain why the effect of litter quality on SOM is inconsistent. For example, although low-quality litter may be mineralized with low microbial CUE by individual taxa, adaptation at the community level to overcome nutrient limitation may allow for decomposition without a reduction in community-level CUE^42,43^. Microbial residue accumulation in soils may also be partly independent of CUE as production rates can be influenced by other biotic factors such as microbial community interactions^44^. Litter quality may also control MAOM stocks through other microbially-mediated mechanisms such as soil priming, the change in microbial decomposition of SOC in response to fresh C inputs^45–47^. For example, a recent global synthesis found that agricultural soils receiving more low-quality litter exhibited higher positive priming^48^. Decreased soil N availability was strongly associated with the magnitude of priming suggesting accelerated nutrient mining of litter decomposers from SOM in response to low-quality litter input^45,48,49^. Litter quality may thus regulate MAOM stocks through microbial interactions that affect both the formation efficiency of new MAOM and the mineralization rate of pre-existing MAOM. The balance between SOC gain through enhanced formation efficiency and loss through priming is rarely considered but determines the net impact of litter quality on SOC accumulation.

The effect of litter quality on SOM may also vary under differing abiotic conditions, such as soils of varied mineralogy^30^. While soil microbial processes produce organic compounds through microbial anabolism, the soil mineral matrix acts as the long-term control on SOM content and stability in soils. However, not all minerals are equivalent in their ability to stabilise organic C^50^. Variation in mineral specific surface area (SSA) and surface charge influences the adsorption and occlusion of organic molecules, which controls the degree of microbial access to organic matter in soils, while clay minerals (e.g. phyllosilicates such as low-activity kaolinite and high-activity montmorillonite) and metal (oxyhydr)oxides can also catalyse polymerization of organic molecules conferring stability^50–52^. Mineralogy may also control the inherent maximum capacity of soils to store mineral-associated SOC (the C saturation concept) as variation in specific surface area (SSA) controls the finite amount of reactive mineral surfaces available for C retention^53,54^. The C saturation deficit of a given soil (proximity to their maximum carbon-storage capacity) may influence the rate of C accumulation, thus modulating the relationship between litter quality and SOM^31,55^. Elucidating these complex interactions between litter quality, microbial anabolism, and soil mineralogy and their effects on MAOM formation is necessary to predict the impact of land-use and climate-driven plant community shifts on MAOM stocks across global soils.

Here we investigated the interactive effects of litter quality, soil mineralogy and soil microbiology on the formation of MAOM, under highly controlled conditions, to explore the mechanisms behind inconsistent effects of litter quality on SOM. Using a laboratory incubation experiment and ^13^C isotopic labelling, we tested how changes in MAOM formation and priming of pre-existing SOM (present before litter addition) were influenced by high- and low-quality litter inputs and quantified how these processes counterbalance each other to affect net SOC accumulation. We hypothesised that inputs of high-quality litter will lead to a net increase in MAOM-C content relative to low-quality litter (H1). This hypothesis is fundamental to modern conceptual models of SOC cycling and is based on the theory of more efficient transformation of microbial residues into MAOM and less priming of existing C leading to an overall net SOC gain. As the soil mineral matrix may exert more control over MAOM retention than the quality of the organic matter itself, we also manipulated soil mineralogy by changing the predominant clay type using pristine phyllosilicate clays with different specific surface area (SSA) (kaolinite and montmorillonite) and a common Fe-oxide mineral in soil (goethite). We hypothesised that soil mineralogy will be a more important control on the formation of MAOM relative to litter quality (H2). Soils with manipulated mineralogy were combined with isotopically labelled high- and low-quality litters and the movement of litter-derived C (hereafter litter-C) was traced into major SOC pools (MAOM, POM and microbial biomass). We also quantified the cumulative loss of soil and litter-C through microbial respiration and further investigated how microbial taxonomy and physiology linked litter quality to SOC stabilisation by measuring soil microbial communities at early (15 days) and late-stage (126 days) decomposition and microbial CUE.

Here we show that soil mineralogy does act as the principal control on MAOM formation, and that litter quality had no effect on total MAOM-C content. However, high-quality litter formed less new MAOM, and its formation was less (not more) efficient. This was associated with shifts in bacterial and fungal community composition, higher 16S ribosomal RNA copy number (a bacterial trait related to growth rate and efficiency), abundance of generalist soil saprotrophs and lower microbial CUE at early-stage decomposition. We propose that high-quality litter formed MAOM less efficiently by the selection of fast-growing copiotroph-dominated communities with lower CUE. Addition of low-quality litter enhanced mineralisation of pre-existing SOC (not derived from added litter), counterbalancing the positive effect of low-quality litter on the efficiency of MAOM formation. This demonstrates how microbial and mineral interactions decouple litter quality from SOC formation.

## Results

### Litter and soil-derived C loss

**Fig. 1:**
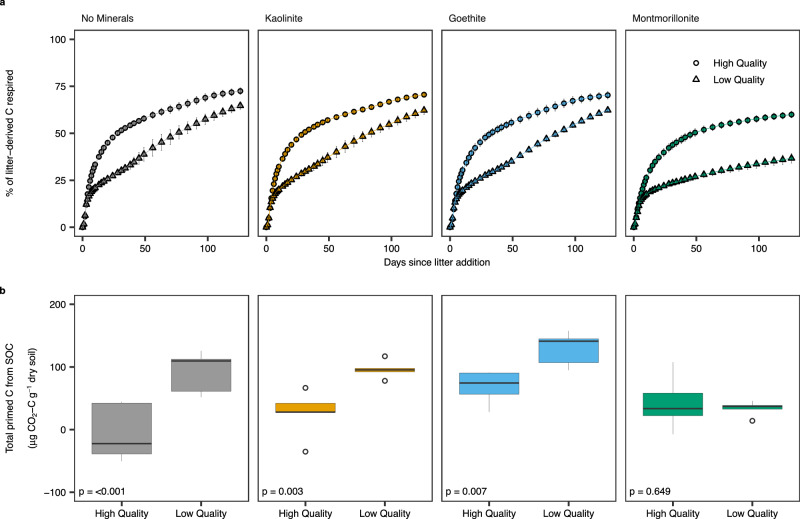
Interactions between litter quality and soil mineralogy determine the amount of carbon (C) respired from added litter and primed from pre-existing soil organic matter (SOM). Cumulative sum of litter-C respired (**a**) and total SOM derived (primed) C (**b**) over the course of the 126-day incubation. CO_2_ sampling was conducted daily from days 0-10 and then on days 13, 15, 17, 20, 24, 28, 31, 34, 38, 41, 45, 49, 56, 63, 70, 77, 84, 91, 98, 105, 112, 119 and 126. The cumulative sum of litter-C respired is presented as a percentage of the C added as litter whilst primed C represents pre-existing C respired from litter addition treatments in excess of C respired from no litter control microcosms. Cumulative litter-C respired data are presented as mean values (*n* = 5) ± 1 standard deviation (displayed as error bars). Boxplot centre lines represent the median, box limits indicate the first and third quartiles, whiskers extend to the minimum and maximum values within 1.5x of the interquartile range and circles represent outliers (defined as points >1.5 times the interquartile range). All statistics were derived from *n* = 5 independent samples. Between group differences in primed C were compared using estimated marginal means (emmeans) to determine the effect of litter quality by mineral treatment. The two-sided *p*-values from emmeans tests are adjusted for multiple comparisons using the Benjamini–Hochberg (BH) procedure (displayed within the plots). The sample size ‘*n*’ represents samples taken from independent experimental units (soil incubations). Source data are provided as a Source Data file. Exact *P*-values are available in the corresponding Source Data file.

**Fig. 2:**
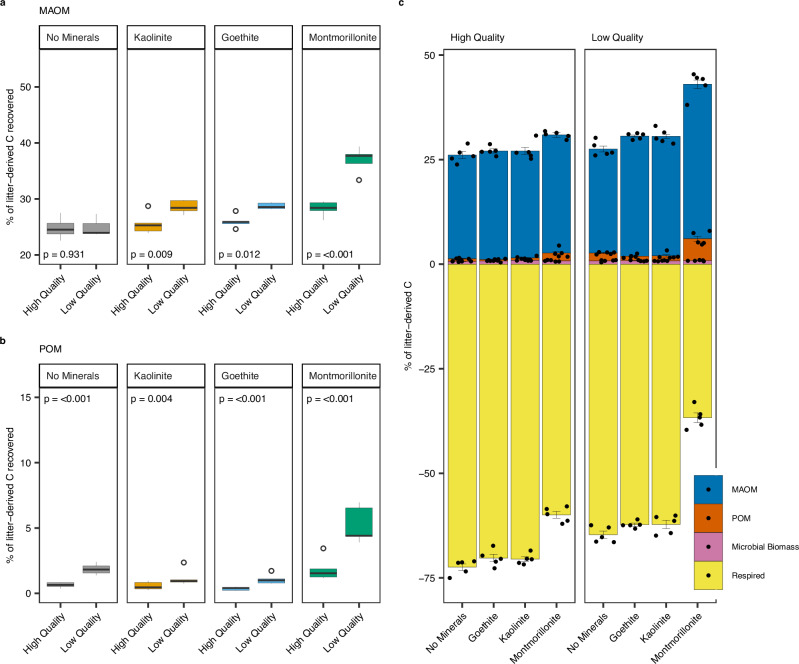
The fate of carbon (C) across major soil organic carbon (SOC) pools is determined by interactions between litter quality and soil mineralogy with low-quality litter having the greatest potential for stabilisation. Percentage of initial litter-C recovered in mineral-associated organic matter (MAOM) (**a**), particulate organic matter (POM) (**b**) and the litter-C distribution between measured C pools (**c**). All data shown are for soils destructively harvested at the end of the 126-day incubation (T126). All C pools were independently quantified using soil fractionation (MAOM, POM), extraction (microbial biomass) and cumulative headspace measurement (respired). Boxplot centre lines represent the median, box limits indicate the first and third quartiles, whiskers extend to the minimum and maximum values within 1.5x of the interquartile range and circles represent outliers (defined as points >1.5 times the interquartile range). In panel c, bars represent data means (*n* = 5) ± 1 standard error (displayed as error bars) and raw data are overlaid to show the underlying data distribution. Litter C respired from incubations and recovered in soil pools are presented as negative (C loss) and positive (C retention) values respectively. All statistics were derived from *n* = 5 independent samples. Between group differences in litter-C recovered in MAOM and POM were compared using estimated marginal means (emmeans) to determine the effect of litter quality by mineral treatment. The two-sided *p*-values from emmeans tests are adjusted for multiple comparisons using the Benjamini–Hochberg (BH) procedure (displayed in the plots). The sample size ‘*n*’ represents samples taken from independent experimental units (soil incubations). Source data are provided as a Source Data file. Exact *P*-values are available in the corresponding Source Data file.

We manipulated an agricultural sandy loam soil (Table 1) with the pristine phyllosilicate clay minerals kaolinite (low-activity clay), montmorillonite (high-activity clay) and a common Fe-oxide mineral found in soils (goethite) to create soils with distinct mineralogy and contrasting in soil mineral reactivity as defined by SSA and charge (kaolinite: low SSA, negative charge; montmorillonite: high SSA, negative charge; goethite: low SSA, variable charge). We assessed the effects of litter quality on MAOM formation by addition of low and high-quality litter (Table 1) to manipulated soils and a “no minerals” control soil to test our primary hypothesis that high-quality litter promotes MAOM formation. Respiration of soil and litter-C was monitored continuously over 126 days until microcosms were destructively harvested. Our results showed that more high-quality litter-C was respired relative to low-quality litter across all soils (Figs. 1a and 2c). Soil mineralogy was the strongest control on respired litter-C (57.2% of total variability explained) with the magnitude of the litter effect controlled by soil mineralogy (Supplementary Table 1; *p* < 0.001 mineral*litter). This interaction was driven by the bigger difference in respired litter-C between litters in high-activity, montmorillonite clay soils (Fig. 1a). Cumulative primed C (difference in soil-derived CO_2_-C emissions in litter vs no litter soils) was controlled by litter quality although the magnitude of the litter quality effect varied across soil mineralogy treatments (Supplementary Table 1; *p* = 0.006 mineral*litter) (Fig. 1b). Pre-existing SOC mineralisation was enhanced by low-quality litter in no mineral, kaolinite, and goethite soils but there was no difference between litters in montmorillonite soils (Fig. 1b).

**Table 1:** Initial soil properties prior to mineral amendment and plant litter chemistry

	Total C (%)	Total N (%)	C:N	Bulk Density (g cm^−3^)	pH	δ^13^C	Atom % ^13^C
Soil	2.25 (0.04)	0.20 ( < 0.01)	11.41 (0.11)	1.43 (0.08)	5.72 (0.02)	-28.54 (0.16)	1.080 (<0.001)
Low Quality Litter	41.33 (0.12)	0.92 (0.02)	44.77 (2.09)	-	-	1348.85 (3.89)	2.572 (0.004)
High Quality Litter	39.45 (0.49)	2.36 (0.02)	16.69 (0.18)	-	-	1414.66 (3.94)	2.642 (0.004)

Data are means (*n* = 5 in each treatment) with the standard error in parentheses.

### Litter quality and soil mineralogy controls on the fate of new C inputs to soils

At the end of the incubation 22.6-39.3% of added litter-C was recovered in the MAOM pool (Fig. 2a and c). Soil mineralogy was the strongest control on the amount of litter-C stabilised as MAOM, explaining 51.4% of the variation with the effect of litter quality on litter-C stabilised as MAOM controlled by soil mineralogy (Supplementary Table 2; *p* < 0.001, mineral*litter). More litter-C was stabilised as MAOM from low- relative to high-quality litter across all soil mineral treatments (Montmorillonite: +8.7%, Goethite: +2.8%, Kaolinite: +3.0% litter-C recovered), with the greatest difference in montmorillonite soils (Fig. 2a and c). In soils without mineral amendment, the amount of litter-C stabilised as MAOM did not vary with litter quality (Fig. 2a and c).

A smaller proportion of added litter-C was recovered as POM (0.2–7.0%) (Fig. 2b, c). Litter quality explained 33.5% of the variation in litter-C recovered as POM while soil mineralogy explained 49.7% (Supplementary Table 2). Across all mineral treatments, more C from low-quality litter was recovered as POM than from high-quality litter (Fig. 2b, c). More C from low-quality litter was also assimilated into microbial biomass relative to high-quality litter during early-stage decomposition (0.56-3.83%) (Supplementary Table 3). The proportion of litter-C recovered in microbial biomass was lower at late-stage decomposition (0.39-1.03%) and did not vary between mineral treatments (Fig. 2c).

**Fig. 3:**
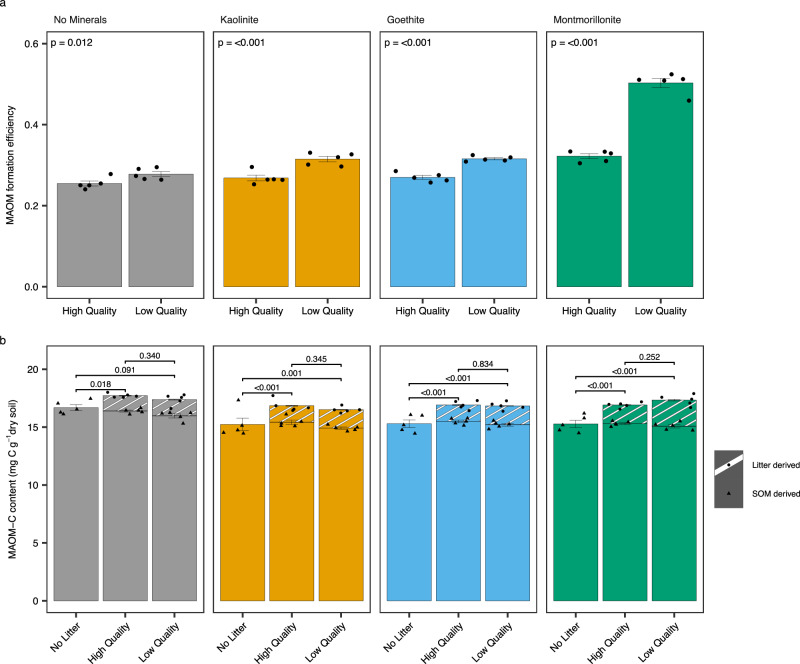
Variation in formation efficiency and total mineral-associated organic matter (MAOM) carbon (C) content is dependent on soil mineralogy and litter quality. MAOM formation efficiency (**a**) and total MAOM-C content partitioned into soil organic matter and litter-derived components (**b**). MAOM formation efficiency was calculated as MAOM-C_litter_ / (MAOM-C_litter_ + Respired C_litter_) to account for the different decomposition rates of low- and high-quality litter. Bars indicate data means (*n* = 5) ± 1 standard error (displayed as error bars). Raw data are overlaid on bar charts to show the underlying data distribution. All statistics were derived from *n* = 5 independent samples. Between group differences in MAOM formation efficiency and total MAOM-C content were compared using estimated marginal means (emmeans) to determine the effect of litter quality by mineral treatment. The two-sided *p*-values from emmeans tests are adjusted for multiple comparisons using the Benjamini–Hochberg (BH) procedure (displayed in the plots). The sample size ‘*n*’ represents samples taken from independent experimental units (soil incubations). Source data are provided as a Source Data file. Exact *P*-values are available in the corresponding Source Data file.

To account for the different decomposition rates of low- and high-quality litter (Fig. 1a) an index of MAOM formation efficiency was calculated (MAOM Formation Efficiency = MAOM-C_litter_ / (MAOM-C_litter_ + Respired C_litter_) where MAOM-C_litter_ is the total litter-C recovered in the MAOM fraction and Respired C_litter_ is the litter-C respired throughout the experiment). Soil mineralogy was the dominant control on MAOM formation efficiency (57.3% of total variability explained) with litter-C stabilised as MAOM most efficiently in high-activity montmorillonite clay soils. In all soils low-quality litter formed new MAOM more efficiently than high-quality litter (Fig. 3a) but the effect of litter quality was larger for montmorillonite soils (Supplementary Table 4; *p* < 0.001, mineral*litter). Litter addition increased the total MAOM-C content in microcosms (including C derived from litter additions and pre-existing SOC present before litter addition) relative to no litter controls (Fig. 3b). However, microcosm total MAOM-C content did not differ between low- and high-quality litter (Fig. 3b, Supplementary Table 4). This indicates that more pre-existing MAOM-C was lost in low- relative to high-quality litter treatments, counterbalancing the effect of litter quality on litter-C transfer to MAOM (Fig. 3b, Supplementary Table 4). This is partially supported by increased priming of pre-existing SOC in low- relative to high-quality litter treatments for no minerals, kaolinite, and goethite soils (Fig. 1b).

### Microbial and mineral impacts on the efficiency of new MAOM formation

Litter additions significantly increased the total amount of microbial biomass in soils at early (T15) and late-stage (T126) decomposition (Table 2, Supplementary Table 5). Averaged across soils, total microbial biomass carbon was greater in high- relative to low-quality litter treatments during early-stage decomposition (*p* = 0.02). However, there was no difference in microbial biomass carbon between the two litter treatments at late-stage decomposition (*p* = 0.98) (Supplementary Table 5).

**Table 2:** Microbial metrics and microcosm soil pH at early (T15) and late-stage decomposition (T126)

Timepoint	Soil	Litter	Soil pH	MBC (µg g^−1^ dry soil)	CUE	Bacterial Richness	rRNA copy number	Fungal Richness
T15	No Minerals	No Litter	5.88 (0.01)	60.92 (0.93)	-	247 (33)	2.29 (0.09)	444 (40)
T15	Kaolinite	No Litter	5.81 (0.03)	54.84 (0.35)	-	264 (31)	2.39 (0.03)	438 (34)
T15	Goethite	No Litter	5.59 (0.04)	59.36 (1.49)	-	240 (28)	2.41 (0.04)	431 (21)
T15	Montmorillonite	No Litter	6.54 (0.03)	65.15 (3.39)	-	290 (25)	2.41 (0.04)	409 (22)
T15	No Minerals	Low Quality	6.51 (0.05)	135.90 (5.87)	0.06 (0.01)	264 (26)	3.14 (0.09)	280 (24)
T15	Kaolinite	Low Quality	6.52 (0.07)	137.35 (6.17)	0.06 (0.00)	287 (21)	3.04 (0.15)	262 (15)
T15	Goethite	Low Quality	6.30 (0.01)	131.53 (4.51)	0.10 (0.01)	248 (43)	2.84 (0.07)	199 (28)
T15	Montmorillonite	Low Quality	7.07 (0.03)	122.19 (5.46)	0.06 (0.01)	303 (16)	3.27 (0.11)	299 (12)
T15	No Minerals	High Quality	6.15 (0.02)	143.28 (20.41)	0.03 (0.00)	303 (25)	3.01 (0.06)	254 (8)
T15	Kaolinite	High Quality	6.12 (0.01)	151.42 (6.26)	0.03 (0.00)	300 (33)	2.92 (0.08)	237 (22)
T15	Goethite	High Quality	5.81 (0.01)	192.20 (23.83)	0.03 (0.00)	310 (10)	3.08 (0.13)	224 (13)
T15	Montmorillonite	High Quality	6.83 (0.01)	150.22 (11.11)	0.03 (0.00)	278 (24)	3.71 (0.07)	220 (7)
T126	No Minerals	No Litter	5.63 (0.01)	52.89 (1.96)	-	298 (11)	2.48 (0.02)	384 (18)
T126	Kaolinite	No Litter	5.67 (0.01)	49.73 (1.70)	-	348 (53)	2.49 (0.01)	390 (17)
T126	Goethite	No Litter	5.42 (0.04)	44.22 (0.75)	-	302 (23)	2.45 (0.01)	399 (13)
T126	Montmorillonite	No Litter	6.79 (0.01)	38.75 (7.98)	-	305 (12)	2.43 (0.02)	355 (15)
T126	No Minerals	Low Quality	6.18 (0.04)	82.92 (12.54)	-	345 (11)	2.44 (0.02)	251 (6)
T126	Kaolinite	Low Quality	6.25 (0.05)	78.91 (3.92)	-	337 (11)	2.37 (0.02)	242 (9)
T126	Goethite	Low Quality	5.9 (0.02)	88.88 (7.01)	-	331 (32)	2.4 (0.03)	260 (21)
T126	Montmorillonite	Low Quality	7.04 (0.05)	84.40 (6.78)	-	317 (15)	2.63 (0.03)	300 (6)
T126	No Minerals	High Quality	5.65 (0.01)	78.07 (2.38)	-	325 (37)	2.56 (0.04)	226 (23)
T126	Kaolinite	High Quality	5.82 (0.02)	95.25 (6.45)	-	322 (17)	2.56 (0.02)	224 (12)
T126	Goethite	High Quality	5.64 (0.03)	81.52 (10.75)	-	314 (33)	2.54 (0.02)	205 (19)
T126	Montmorillonite	High Quality	7.29 (0.06)	83.33 (7.71)	-	314 (7)	2.79 (0.02)	261 (7)

Carbon use efficiency (CUE) was calculated for T15 only as using ^13^C to estimate CUE is sensitive to incubation time and is known to decrease over long-term incubations because of microbial turnover. Data are means (*n* = 5 in each treatment) with the standard error in parentheses.

**Fig. 4:**
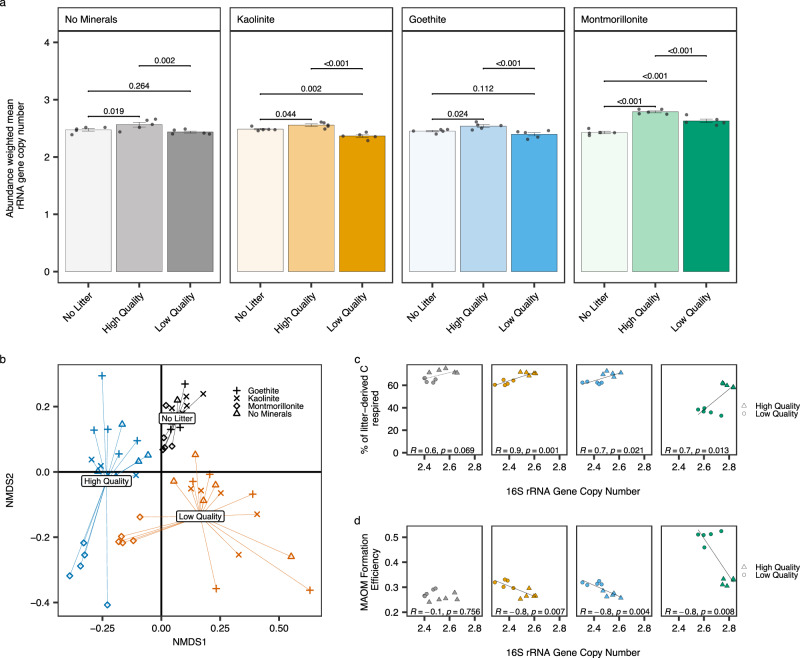
Effects of litter quality and soil mineralogy on soil bacterial communities and relationship to litter carbon (C) stabilisation and loss. Abundance-weighted mean 16S rRNA gene copy number for each treatment (**a**), Soil bacterial community composition (**b**), relationship between abundance-weighted mean 16S rRNA gene copy number and percentage of litter-derived C respired (**c**) and the efficiency of mineral-associated organic matter (MAOM) formation (**d**). In (**a**), bars indicate data means (*n* = 5) ± 1 standard error (displayed as error bars). Raw data are overlaid on bar charts to show the underlying data distribution. Bacterial communities from the end of the 126 day incubation (T126) are presented in (**b**) using non-metric multidimensional scaling analysis conducted on a Bray-Curtis dissimilarity matrix demonstrating strong impacts of the litter (labelled and colour-coded spiders) and mineral (indicated by symbol shape) treatments. All statistics were derived from *n* = 5 independent samples. Between group differences in abundance-weighted mean 16S rRNA gene copy number were compared using estimated marginal means (emmeans) to determine the effect of litter quality by mineral treatment. The two-sided *p*-values from emmeans tests are adjusted for multiple comparisons using the Benjamini–Hochberg (BH) procedure. Solid and dashed lines in (**c**, **d**) represent two-sided pearson correlations at *p* < 0.05 and *p* < 0.1 respectively. *P*-values derived from correlations were not adjusted for multiple comparisons. The sample size ‘*n*’ represents samples taken from independent experimental units (soil incubations). Source data are provided as a Source Data file. Exact *P*-values are available in the corresponding Source Data file.

Soil bacterial community samples clustered strongly by litter indicating that litter quality shifted soil bacterial community composition at both early (Supplementary Fig. 1a, Supplementary Table 6) and late-stage decomposition (Fig. 4b, Supplementary Table 6). These bacterial community shifts were associated with changes in the number of ribosomal RNA operons in bacterial genomes (hereafter referred to as copy number) (Fig. 4a, Supplementary Table 6). The copy number predicts maximum bacterial growth rate and growth efficiency^56,57^. Maximum growth rate increases with an increase in copy number while bacterial carbon use efficiency is inversely related to copy number and maximum growth rate. Litter addition increased copy number during early-stage decomposition relative to no litter controls (Supplementary Fig. 1b, Table 2, Supplementary Table 6). Copy number did not differ with litter quality in no mineral, kaolinite, and goethite soils (Supplementary Fig. 1b). However, copy number was greater in high- relative to low-quality litter in montmorillonite soil (Supplementary Fig. 1b). At late-stage decomposition copy number was greater in high- relative to low quality litter treatments in all soils (Fig. 4a) and was positively correlated with total litter-C respired in all mineral amended soils (Fig. 4c). MAOM formation efficiency was negatively correlated with copy number in all mineral amended soils (Fig. 4d) but in soils without mineral amendment, the litter-C respired and MAOM formation efficiency were not associated with copy number (Fig. 4c, d).

**Fig. 5:**
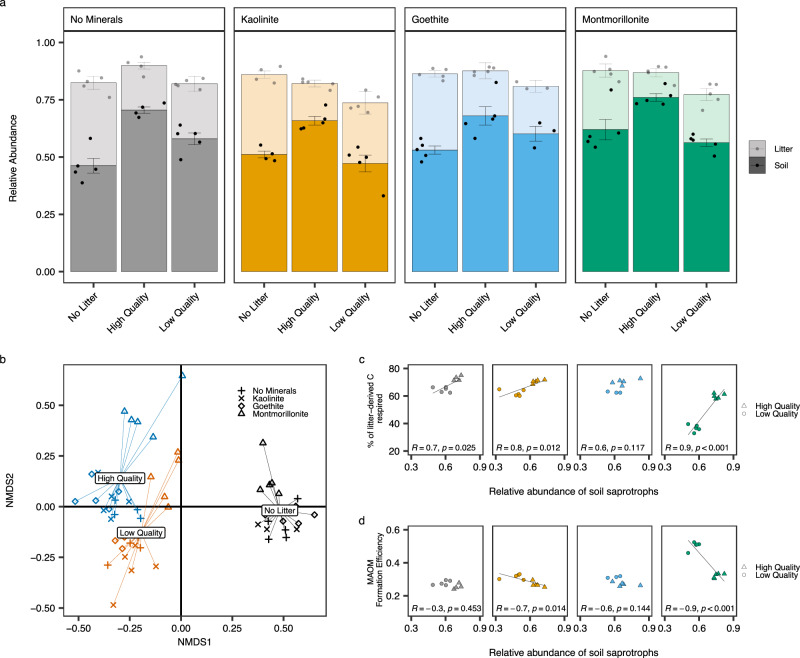
Effects of litter quality and soil mineralogy on soil fungal communities and relationship to litter carbon (C) stabilisation and loss. Relative abundance of fungal saprotrophs partitioned into specialist litter and generalist soil saprotrophs (**a**), Soil fungal community composition (**b**), relationship between soil saprotrophs and percentage of litter-derived C respired (**c**) and the efficiency of mineral-associated organic matter (MAOM) formation (**d**). 4 samples were excluded due to a low number of sequence reads (1 sample from No Minerals: High Quality, Kaolinite: No Litter and 2 samples from Goethite: Low Quality treatments). In panel a, bars indicate data means (*n* = 5 independent samples) ± 1 standard error (displayed as error bars) (*n* = 4 for No Minerals: High Quality and Kaolinite: No Litter treatments. *n* = 3 for Goethite: Low Quality treatment). Raw data are overlaid on bar charts to show the underlying data distribution. In panel b fungal communities from the end of the 126 day incubation (T126) are presented using non-metric multidimensional scaling analysis conducted on a Bray-Curtis dissimilarity matrix demonstrating strong impacts of the litter (labelled and colour-coded spiders) and mineral (indicated by symbol shape) treatments. Statistics in panel c and d were derived from *n* = 5 independent samples (*n* = 4 for No Minerals: High Quality treatment. *n* = 3 for Goethite: Low Quality treatment). Solid lines represent two-sided pearson correlations at *p* < 0.05. *P*-values derived from correlations were not adjusted for multiple comparisons. The sample size ‘*n*’ represe*n*ts samples taken from independent experimental units (soil incubations). Source data are provided as a Source Data file. Exact *P*-values are available in the corresponding Source Data file.

Litter addition reduced fungal richness (Table 2) at early and late-stage decomposition relative to no litter controls, but richness did not differ with litter quality. Soil fungal communities were also strongly clustered by litter indicating that litter quality was a strong control on fungal community composition at early- and late-stage decomposition (Supplementary Fig. 2b, Fig. 5b, Supplementary Table 7). At early-stage decomposition the relative abundance of generalist soil saprotrophs and specialist litter saprotrophs were both strongly influenced by litter quality (Supplementary Table 7, Supplementary Fig. 2a). Within each soil, the relative abundance of generalist soil saprotrophs was positively correlated with litter-C respired, driven by higher abundance in high- relative to low-quality litter treatments (Supplementary Fig. 2c). Conversely, the relative abundance of specialist litter saprotrophs was negatively correlated with litter-C respired due to higher abundance in low- relative to high-quality litter treatments (Supplementary Fig. 2d). Soil mineralogy also controlled the relative abundance of specialist litter saprotrophs with lower abundance in montmorillonite soils (Supplementary Table 7, Supplementary Fig. 2a). Across soils (within each litter addition treatment), specialist litter saprotroph abundance was positively associated with the amount of litter-C respired (Supplementary Fig. 3). At late-stage decomposition, the relative abundance of generalist soil saprotrophs was still greater in soils with high- compared to low-quality litter, across all mineral treatments (Fig. 5a) and were dominated by fast-growing generalist taxa from the genera *Mortierella* (Supplementary Fig. 4). Soils with low-quality litter added were characterised by higher relative abundance of litter saprotrophs (Fig. 5a) including ascomycetous molds (*Cladosporium*) (Supplementary Fig. 5). Total litter-C respired was positively correlated with the relative abundance of soil saprotrophs in no mineral, kaolinite and montmorillonite soils whilst MAOM formation efficiency was negatively correlated (Fig. 5c, d). In goethite soils, total litter-C respired and MAOM formation efficiency was not associated with the abundance of soil saprotrophs (Fig. 5c, d).

## Discussion

Contrary to our first hypothesis that the addition of high-quality litter would lead to more efficient MAOM formation (H1), we observed the opposite where low-quality litter led to greater and more efficient formation of litter-C stabilised as MAOM across all soils (Figs. 2, 3). This is despite high-quality litter degrading faster than low-quality litter and contrasts with contemporary SOC theory that suggests high-quality litter forms more MAOM^16,34,37–39,58^. A small number of recent isotope tracing studies that evaluated litters with contrasting chemistry do support our findings^31,41,59^. Córdova, Olk^31^ also found that whilst high-quality litter formed more MAOM, its formation was less efficient relative to low quality litter. However, these studies did not measure microbial community responses to litter additions. A recent meta-analysis also revealed that slow rather than fast decomposing litter led to more efficient transfer of C to SOC, although this analysis did not differentiate between C fractions (MAOM and POM) or detail possible mechanisms for this effect of litter quality^32^. Low quality litter environments typically form more POM due to slower decomposition rates, promoting physical transfer of plant material through the soil profile^15,16,60^. In agreement, we found that more litter-C was recovered as POM from soils amended with low-relative to high-quality litter. However, the proportion of litter-C recovered as POM was low relative to MAOM (Fig. 2c).

Our hypothesis was derived from the expectation that microbes would partition more C used in metabolism towards growth versus respiration upon decomposition of high- relative to low-quality substrates as previously observed^28,61^. However, while substrate chemistry can alter microbial CUE due to direct impacts on cellular metabolism, it may also drive CUE indirectly, by the selection of distinct microbial communities with different prevailing life histories^62^. For example, simple C substrates typically promote higher microbial CUE within a community but over time may also select for a copiotroph-dominated community with inherently lower CUE^63^. We observed strong litter-associated shifts in microbial community composition (Figs. 4b, 5b), differences in the bacterial copy number (a functional trait associated with growth rate and efficiency) (Fig. 4a, Supplementary Fig. 1b), and that relative abundance of generalist soil fungal saprotrophs was negatively correlated with MAOM formation efficiency (Fig. 5d). We also found that high-quality litter addition reduced microbial CUE during early-stage decomposition (Table 2) as in soils with high-quality litter microbial CUE was approximately half that of the low-quality litter treatments (average of 0.03 and 0.07 respectively; Table 2). This suggests that although high-quality litter may be processed with higher CUE, this effect is outweighed by a community-level selection towards taxa with life history traits that promote growth rate at the expense of CUE. We caution that the use of ^13^C litter tracing to calculate CUE will likely reflect a subset of the microbial community active on the litter substrate and declines with incubation duration due primarily to increased cumulative respiration of ^13^C^64^. We chose a longer incubation duration (15 days) to integrate the effects of shifts in microbial community composition in response to litter inputs resulting in low CUE values. However, we acknowledge that this long incubation potentially also includes the effect of turnover and substrate recycling and make no comparison to other published CUE estimates^64^. Nonetheless these findings are contradictory to the expectation that a labile C environment would increase population-level microbial CUE and therefore generate more microbially-derived SOM^26,65,66^. Litter chemistry thus appears to be less important for SOM accumulation than how it interacts with microbial communities but further supports the premise that microbial CUE is a major, although indirect, determinant of global soil C storage^67^.

A second mechanism that could decouple litter quality from MAOM accumulation is enhanced SOM mineralisation (e.g. priming effects) following fresh litter addition^68^. We hypothesised that high-quality litter would cause less priming of existing SOM relative to low-quality litter due to less microbial N mining from existing MAOM (H1). In partial agreement, low-quality litter caused more priming of native SOM than high-quality litter across no mineral, kaolinite, and goethite soils (Fig. 1b). This was offset by the enhanced MAOM formation efficiency observed from low-quality litter resulting in equal total MAOM-C content across litter treatments (Fig. 3a, b). This may be due to microbial N mining via the decomposition of native SOM, resulting in stronger priming effects following low-quality litter addition^45,46,68^. The C:N ratio of MAOM was higher in low- relative to high-quality litter which does support the N mining hypothesis (Supplementary Table 8). It may also reflect observed litter-associated microbial community responses as the intensity of priming may depend on the type of microbial populations stimulated by litter addition^69^. Others have shown that the addition of complex leaf litter causes a higher bacterial oligotroph/copiotroph (K-/r-strategists) ratio, enhancing SOM decomposing enzyme activity and leading to a far greater priming effect than when simple substrates were added^70,71^. However, there was no effect of litter quality on priming in montmorillonite soils. This suggests that variation in the soil mineral matrix could also directly (variation in soil mineral SSA) or indirectly (mineral effects on soil pH or moisture) override the effect of microbial physiological traits on MAOM.

In agreement with our second hypothesis, that soil mineralogy would control the formation of MAOM more than litter quality (H2), we found that soil mineralogy explained most variation in litter-C respired (57.2%) and litter-C stabilised in MAOM (51.4%), whilst the effect of litter type was smaller (20.8%) and dependent on soil mineralogy (Fig. 1a, Fig. 2a, Supplementary Table 1 and 2). This suggests that a significant proportion of plant C compounds may have been directly stabilised through sorption. However, it could also reflect microbe-mineral interactions that shifted microbial community composition and physiology, promoting biomass production (efficiency) and stabilization of resultant microbial products by association with soil minerals. We did not measure biomarkers in MAOM to quantify plant and microbial derived SOM and there are known limitations with these methods^72^. However, a recent meta-analysis suggested that whilst microbial pathways of SOM formation are important, ~50% of MAOM-C may be plant-derived^25^. Therefore, improving quantification of plant and microbially derived SOM is a key research need^72^.

Although the effect of mineralogy on SOC dynamics is known as a key determinant of soil C stocks^73^, this study demonstrates how mineralogy and litter quality interact with soil microbes to control the efficiency of MAOM formation. Soils amended with montmorillonite had a greater capacity to form MAOM and it was formed more efficiently, from both litter types, relative to kaolinite (Fig. 3). This agrees with other studies of organo-mineral formation where artificial soils were created with pristine minerals^74,75^ and is also consistent with the greater adsorption capacity of 2:1 vs 1:1 phyllosilicates. 2:1 phyllosilicates have higher specific surface area (SSA) and more frequent isomorphic cation substitutions relative to 1:1 phyllosilicates, leading to negative surface charge^76,77^. This promotes the adsorption of organic molecules through cation bridging^51^. We acknowledge that our results may be influenced by applying pristine minerals to soils, as most mineral surfaces in natural systems are already associated with organic molecules. However, a study by Pronk, Heister^78^ using artificial soils found substantial sorption of organic matter to fresh mineral surfaces within a few days. As pristine minerals were added to a natural soil, organo-mineral associations may have formed rapidly with pre-existing SOM prior to the start of the experiment. These organo-minerals may more closely resemble mineral particles found in real soils. Nonetheless, caution should be exercised when translating these findings to natural systems. The finding that montmorillonite soils formed new MAOM from plant litter more efficiently than kaolinite soils may also be due to a greater C saturation deficit (due to different SSA of pristine minerals added to soils) as MAOM exhibits saturation dynamics due to the finite availability of mineral surfaces^79^. Our findings agree with previous work showing that MAOM formation efficiency is strongly governed by the soil C saturation status with the highest efficiency at the greatest C saturation deficit^80–82^. It has also been proposed that in soils with high C saturation deficits the conversion rate of litter-C to mineral-associated SOC is high, regardless of litter quality^30^. However, we observed the greatest litter quality effect in montmorillonite amended soils with the greatest C saturation deficit.

Although litter quality explained most variation in soil microbial communities, the effect of mineralogy was greatest in montmorillonite soils (Supplementary Table 6 and 7). We propose that this is likely due to microbial-mineral interactions as growth of microbial taxa may vary among soils of different mineral assemblages^83^, but may also reflect a response to mineral-mediated changes in abiotic soil properties such as pH and moisture retention. However, a key uncertainty remains concerning the relative importance of mineral chemistry itself, against indirect mineral effects on the soil abiotic environment in shaping microbial community responses. Nonetheless, our findings suggest that the effect of mineralogy on SOM dynamics may not be solely abiotic, and that microbe-mineral interactions could modify the effect of litter quality on MAOM formation efficiency.

There was no difference in the MAOM formation efficiency between kaolinite and goethite soils (Fig. 3a). This was surprising as Fe-oxides (especially non-crystalline ferrihydrite) are highly reactive in soils^84^ and the addition of goethite to soils has been shown to promote organic matter accumulation^85,86^. However, we used a commercially prepared goethite (SSA: 11.18 ± 0.07) (Sigma Aldrich), comparable in SSA to kaolinite (SSA: 10.05 ± 0.02). Moreover, we added solid-phase goethite under controlled moisture conditions, restricting the mechanism of SOC retention to adsorption, rather than dissolution and re-precipitation^87^, which can lead to higher C retention on goethite^88^.

Our controlled laboratory incubation of amended soils does not include the full range of processes that occur under real-world conditions. For example, litter quality is also confounded with variation in production rates, which may lead to different soil microenvironments. However, our results suggest that inconsistency in the effect of litter quality on MAOM accumulation is driven by differences in the soil mineral matrix; microbial responses to litter additions; and priming of pre-existing SOM. In this experiment the soil mineral matrix was of primary importance, influencing the stabilisation of newly formed MAOM whilst also controlling the magnitude of primed C from pre-existing SOM following litter additions. Strong microbial community responses to litter addition were associated with the formation efficiency of new MAOM such that, contrary to our current understanding low-quality litter formed new MAOM most efficiently. However, this was offset by higher priming of pre-existing SOM, resulting in no net difference in MAOM-C content. Our results suggest that high-quality litter does not always enhance total SOC stocks because of microbial interactions, and a more holistic view of the links between plant inputs, soil microbes and soil minerals is required that can be represented in the next generation of SOC models. Resolving the key fundamental mechanisms that control SOM persistence is crucial to optimise land management that can maintain or increase SOC stocks for climate change mitigation and predict soil C feedbacks to climate change.

## Methods

### Preparation of ^13^C-labelled litter

Litter quality was defined in this study by the C:N ratio, which is predictive of the mineralization rate of litter. To trace litter-C into soils, a 2.5716 atom% ^13^C-labelled winter wheat (*Triticum aestivum*) litter (high C:N ratio; low-quality) and a 2.6417 atom% ^13^C white clover *(Trifolium repens)* litter (low C:N ratio, high-quality) were prepared in-house. Seeds were sown into 8 cm pots filled with a 1:1 quartz sand and perlite mix. Upon germination, pots were placed inside a 900 × 900 x 900 mm Perspex labelling chamber with a removable lid. The labelling chamber was sealed daily for 1 h and 50 ml of 99 atom% ^13^CO_2_ was injected periodically in 10 ml increments and fixed into plant tissues. Plants also received weekly nutrient additions of a 50% strength Hoagland’s solution. The aboveground litter was grown for 16 weeks under ambient environmental conditions and then harvested. Litter was oven-dried at 50 °C, milled and screened through a 250 µm mesh. A subsample was pulverised and analysed for C, N and δ^13^C using methods described below.

### Soil Collection and Analysis

Topsoil for the incubation (0-15 cm) was collected during August 2020 from an agricultural field used for silage production near Glassonby, Penrith, northern England, using 40 plastic PVC cores (5.1 cm internal diameter, 15 cm depth). This soil was chosen due to its coarse texture (sandy loam: 9% clay, 24% silt, 67% sand), which enabled the bulk manipulation of the clay fraction with pristine minerals. Fresh soil from all collected cores was passed through a 4 mm sieve, homogenised by hand-mixing, and characterised for pH, total C and N, δ^13^C and moisture content. Soil pH was measured on fresh soil subsamples. These were sieved to 4 mm and then 10 g soil was mixed into a slurry with 25 ml deionized water and left to stand for 30 min. The pH of the suspension was then measured using a calibrated pH probe (Hanna pH210 Meter, Hanna Instruments Ltd, UK). A further subsample of soil was sieved to 2 mm, oven-dried (105 °C for 24 h) and moisture loss recorded as field soil moisture content. The oven-dried subsample of soil was ground in a ball mill (Fritsch Planetary Mill, Germany) and a 100 mg subsample was used to assess total C and N concentration using an elemental analyser (Leco Truspec Micro, MI, USA). δ^13^C was determined using a Costech ECS-4010 Elemental Analyser (Costech Analytical Technologies, USA) coupled to a calibrated (cane sugar ( − 11.64 ‰) and beet sugar ( − 26.03 ‰) (IsoAnalytical, UK)) CRDS Picarro G-2131i isotopic analyser (Picarro Inc. CA, USA) via a split-flow interface^89^. Total Inorganic Carbon was quantified using Thermogravimetric analysis (LECO 700, MI, USA). No significant inorganic carbon was present in control or mineral amended soils (0.05-0.06%). Maximum soil water holding capacity was measured on five soil replicates; calculated as the amount of water remaining in the soil after being saturated and left to drain for 24 h in a fully humid airspace^90^. δ^13^C and water holding capacity was also measured on amended soils to account for the effect of mineral addition (See Methods in section 2.3). Field bulk density was calculated from the dry soil weight and volume of three additional replicate cores after accounting for mass and volume of stones^91^.

### Laboratory Incubation

A soil laboratory incubation was conducted under controlled temperature (15 °C) and moisture conditions (65% of maximum soil water holding capacity) to quantify the effects of mineralogy and litter quality on litter-C loss through microbial respiration and incorporation into different SOM fractions. We established twelve treatments: Litter additions of winter wheat (*Triticum aestivum*) and white clover *(Trifolium repens)* and four soil mineralogy treatments (no mineral soil; Low activity clay: Kaolinite; high activity clay: Montmorillonite; Fe-Oxide: Goethite). Microcosms without litter across the four soil treatments served as controls (*n* = 12). Ten replicates were used for each treatment for a total of 120 microcosms. To create the four soil mineralogy treatments, fresh homogenised soil was amended with pristine minerals to create three artificially amended soils in addition to a “no minerals” control soil. Two well characterised clays, Kaolinite (Kga-1b; SSA: 10.05 ± 0.02 m^2^ g^−1^) and montmorillonite (Swy3; SSA: 31.82 ± 0.22 m^2^ g^−1^) were purchased from the Clay Minerals Society Source Clays Repository (Chantilly, VA, USA). Goethite (SSA: 11.18 ± 0.07) was purchased pre-prepared from a commercial supplier (Sigma Aldrich) and the SSA was measured using Brunauer-Emmett-Teller (BET) surface area analysis (N_2_ adsorption) (Gemini VII 2390, Micromeritics Instrument Corporation, USA). To create contrasts in mineralogy and reactivity (defined by SSA and surface charge) as would be expected by changing the predominant clay or mineral type in soil, the three minerals were added to a subsample of the homogenised soil at 10% w/w and soils thoroughly hand homogenised. This approach has been used previously in artificial soils to determine root effects on SOC^85^ and in the context of a real soil system allows the maintenance of a natural soil microbial community within soils that differ in dominant clay minerals. We also measured the SSA of the unamended soil and the final soil-mineral mixtures; The results were as follows: control T0 unamended soil = 3.10 ± 0.01 m2/g; G-amended soil = 4.17 ± 0.02 m2/g; K-amended soil = 4.00 ± 0.02 m2/g; M-amended soil = 3.88 ± 0.01 m2/g. The measured SSA of the M-amended soil is lower than might be expected if a simple mixing of 10% w/w montmorillonite and the control T0 unamended soil (5.97 ± 0.22 m2/g) is assumed. This could reflect occlusion of surfaces by sorption of OM to mineral surfaces following mineral addition or the effects of freeze-thawing and drying on a 2:1 clay vs a 1:1 clay and iron oxide as soils were stored frozen, then thawed and dried for analysis. We therefore consider the SSA of the individual minerals to be more indicative of the reactivity of the soil-mineral mixtures than the above SSA analyses. We chose to use real soils amended with pristine minerals rather than artificial soils to ensure microbial communities were representative of the true complexity found in soils. While artificial soils allow for precise control of soil texture, they necessitate use of a soil wash as an inoculum, which can impose a selection on microbial taxa. Seventy-five g dry weight equivalent soil was added to 1 L Kilner jars and soil moisture content was standardised to 65% of each soil’s maximum water holding capacity (accounting for mineral addition), which was maintained throughout the experiment. Soils were then left to equilibrate at 15 °C for 1 week prior to litter addition. One g aliquots of ^13^C-labelled litter were then surface-applied to soils. Five replicate microcosms were used to assess the short-term microbial response to litter addition during early-stage litter decomposition and destructively harvested after 15 days (T15) while the remaining five replicate microcosms were used for bulk quantification of respired litter-C and transfer to SOM fractions. These were destructively harvested after 126 days of incubation (T126) once litter-C mineralization appeared to stabilise.

### Gas sampling and analysis

Respiration of litter-C was quantified by headspace CO_2_ and ^13^CO_2_ analysis on five replicate microcosms throughout the 126-day duration of the experiment. Immediately following litter addition, jars were flushed using synthetic air (80% N_2_, 20% O_2_, CO_2_-free) for 60 s and sealed with custom lids fitted with butyl rubber septa to facilitate regular headspace gas sampling. Soil respiration was measured daily from days 0-10 and then on days 13, 15, 17, 20, 24, 28, 31, 34, 38, 41, 45, 49, 56, 63, 70, 77, 84, 91, 98, 105, 112, 119 and 126 of the incubation by determining the CO_2_ concentration accumulated in the jar headspace during the time elapsed between the last sampling. After mixing the jar’s headspace with a syringe, a 10 ml subsample was withdrawn and injected into 3 ml pre-evacuated exetainer vials (Labco, Lampeter, UK). A second 30 ml sample was withdrawn and injected into 12 ml pre-evacuated exetainer vials for δ^13^CO_2_ analysis. After each sampling, jars were opened, flushed with synthetic air, resealed and a further gas sample taken to quantify any residual CO_2_ present following flushing.

Concentrations of CO_2_ were analysed on a PerkinElmer Autosystem XL Gas Chromatograph (GC) (PerkinElmer, Waltham, USA) fitted with a Flame Ionisation Detector (FID) operating at 130 °C. The GC was fitted with a stainless steel Porapak Q 50–80 mesh column (length 2 m, outer diameter 3.17 mm) maintained at 60 °C. δ^13^C values of CO_2_ were analysed using a Picarro cavity ring-down spectrometer (model: G2201-I, Picarro, Inc. CA, USA) coupled with a custom-built auto-sampler and are reported relative to the Vienna Pee Dee Belemnite standard. For natural abundance “No Litter” samples the instrument was calibrated for δ^13^C-CO_2_ using isotopic standards with isotope ratios of -7.2 ‰, -13.6 ‰ and -19.4 ‰ and -22.7 ‰ (CK Gases, Leicester, UK). For enriched samples the instrument was calibrated for δ^13^C-CO_2_ using in-house prepared gas standards of 97.7 ‰ and 815 ‰. The overall analytical precision based on replicate measurements of standards was ± 0.3 ‰ for natural abundance and ± 5 ‰ for enriched samples.

### Microcosm soil analysis and organic matter fractionation

Microcosm bulk soils at T15 and T126 were analysed for soil pH, C, N and δ^13^C as described above. SOM fractionation was conducted on soil subsamples taken from the T126 destructive harvest of microcosms using a physicochemical density fractionation method based on the procedure of Schrumpf, Kaiser^92^, which is based on ideas from Golchin, Oades^93^ (Supplementary Fig. 6). We defined POM as material with density <1.8 g cm^−3^ and MAOM as material with density >1.8 g cm^−3^ and particles <53 µm in size according to the operational definitions proposed by Lavallee, Soong^18^. Subsamples (10 g) of sieved, air-dried soil were placed in 100 ml centrifuge tubes with 80 ml sodium polytungstate solution (SPT) (NaPT; Sometu, Belgium) at a density of 1.8 g cm^−3^ and shaken gently for 30 min at 100 rpm on an orbital shaker. Tubes were centrifuged at 1874 g for 30 min and floating material was removed by vacuum filtration onto 0.45 µm cellulose nitrate filters. To remove residual SPT from the free POM fraction, filters were rinsed with milli-Q water. Complete SPT removal was assumed when the conductivity of the rinse water fell below <50 µs cm^−1^ (Measured using a calibrated Jenway 4510 probe). Material was then washed from the filters into individual aluminium foil trays. Liberation of the occluded POM fraction was carried out using ultra sonication (Sonics Vibracell CV18 probe). Another 80 ml SPT was added to each tube and vortexed for 1 min to re-suspend the soil pellet. To minimise the redistribution of C across fractions^94^, the lowest energy required to fully disperse soils was pre-determined by observing the effects of a stepwise increase of sonication energy on liberated light material. Sonication energy was applied in 50 J ml^−1^ increments up to 300 J ml^−1^ and floating light material removed by pipetting prior to each subsequent sonication of 50 J ml^−1^. Complete disruption of aggregates was assumed when no further material was observed floating in the SPT solution. Soils were weakly aggregated as 100 J ml^−1^ was sufficient to fully disperse our soils. Although this sonication energy is low, others have found 100 J ml^−1^ to be sufficient for the dispersal of coarse textured soils^92,95^. Sonication was then performed at a power of 50 W^96^. Tubes were submerged in an ice bath during sonication to maintain sample temperature at <40 °C^92^. Tubes were then centrifuged, material filtered and rinsed with milli-Q as described above. As the mass of occluded POM was generally small, this fraction was combined with the free POM fraction in an aluminium foil tray to form one complete POM fraction, oven-dried at 40 °C and weighed. The centrifuge tubes containing the remaining soil were refilled with milli-Q water and centrifuged at 3871 g for 2 h. This step was repeated four times until the conductivity of the supernatant was <50 µs cm^−1^. The soil pellet was then passed through a 53 µm stainless steel sieve with mechanical agitation to separate the MAOM fraction, transferred to a pre-weighed aluminium tray, oven-dried at 40 °C and weighed. All dried fractions were ground to a fine powder using a Retsch mixer mill (MM400, Retsch, Dusseldorf, Germany) and analysed for C and δ^13^C as described above. Sand particles (>53 µm) retained on the sieve (sometimes termed sand-sized SOM or heavy POM) were also dried, weighed, and analysed for C and δ^13^C but are not discussed.

### Microbial Biomass and community characterisation

Microbial biomass C and soil DNA were extracted and analysed at T15 and T126. Microbial biomass in soils was extracted using a modified chloroform-direct extraction^97^, with a water matrix ( + 1 ml CHCl_3_ in one of the tubes). Extracts were analysed for total extractable carbon and litter-C incorporation using an Aurora 1030 W TOC analyser coupled to a Picarro G2201i CRDS analyzer via a split-flow interface to determine δ^13^C.

DNA was extracted from 0.2 g frozen soil using a Powersoil® DNA Isolation Kit according to the manufacturer’s instructions^98–100^.Bacterial and fungal community compositions were assessed by sequencing the V4-V5 region of the 16S rRNA genes using the 515 f GTGYCAGCMGCCGCGGTAA and 806r GGACTACNVGGGTWTCTAAT primers^101^ and the established primers GTGARTCATCGAATCTTTG and TCCTCCGCTTATTGATATGC coding the ITS2 region^99^. We followed the PCR protocols of the Earth Microbiome Project^102^, using high-fidelity DNA polymerase (Q5 Taq, New England Biolabs). Each amplicon library was sequenced separately using a 2-step Nextera approach on the Illumina MiSeq platform with V2 500 cycle chemistry (Illumina Inc., USA), with 8 pM loads and 7.5% phiX control library.

Sequences were processed using the DADA2 pipeline^100^ in R version 4.3.1 (dada2 package version 1.28.0) to trim, quality-filter, de-noise, de-replicate, generate amplicon sequence variant (ASV) tables, and to assign taxonomies. Briefly, after removal of primer sequences, forward and reverse reads were trimmed to 200 bases, quality thresholds set to maxEE = 1, maxN = 0. All other settings were default. Taxonomic assignment was performed with assignTaxonomy function against UNITE dynamic database 25.07.2023^103^ and the SILVA SSU r132^104^ for fungal and bacterial taxonomic assignment, respectively.

Prior to analysis, amplicon sequences belonging to the genus Thermus (an internal DNA extraction standard) were removed and samples normalized by rarefying to 2888 reads for 16 S and 10984 reads for ITS using the Phyloseq R package (version 1.48.0)^105^. 1 and 6 samples from 16 S and ITS data respectively were excluded from further analysis due to low read counts. Copy numbers of bacterial taxa were predicted using the ANNA16 tool that estimates gene copy number directly from the 16S rRNA gene sequence strings, without resolving taxonomy or phylogeny^106^. Fungal taxa were annotated as saprotrophic using the FungalTraits database^107,108^.

### Data Analysis

To determine the amount of litter-C in bulk soil, soil fractions, microbial biomass and headspace CO_2_ relative to the added litter C addition_,_ a two-component isotope mixing model was used according to the following equation:

1 $${{{\rm{C}}}}_{{{\rm{litter}}}}=\frac{({{{\rm{\delta }}}}^{13}{{{\rm{C}}}}_{{{\rm{microcosm}}}}-\,{{{\rm{\delta }}}}^{13}{{{\rm{C}}}}_{{{\rm{control}}}})}{({{{\rm{\delta }}}}^{13}{{{\rm{C}}}}_{{{\rm{litter}}}}\,-{{{\rm{\delta }}}}^{13}{{{\rm{C}}}}_{{{\rm{control}}}})}$$

where δ^13^C_microcosm_ is the measured δ^13^C of the C pool (bulk soil, soil fractions, microbial biomass, or headspace CO_2_) in the microcosm after destructive harvesting, δ^13^C_control_ is the δ^13^C of the C pool in no litter control treatments and δ^13^C_litter_ is the measured δ^13^C of the added ^13^C-labelled litter (wheat or clover).

This C_litter_ value was multiplied by the mass of C in each pool to calculate the absolute value of the litter-C in each pool and this was further divided by the original addition to calculate the % recovery of ^13^C-labelled litter in each C pool. Percent recovery of litter-C in headspace CO_2_ was calculated by cumulative summing of litter CO_2_-C at each measured timepoint.

For microbial biomass C, the calculation for fraction of litter-C incorporation into the microbial biomass flush was calculated as follows:

2 $${{{\rm{MB}}}{{\rm{\delta }}}}^{13}{{\rm{C}}}=\frac{{{{\rm{MB}}}{{\rm{\delta }}}}^{13}{{{\rm{C}}}}_{{{\rm{fumigated}}}}*{{{\rm{C}}}}_{{{\rm{fumigated}}}}-\,{{{\rm{MB}}}{{\rm{\delta }}}}^{13}{{{\rm{C}}}}_{{{\rm{unfumigated}}}}*\,{{{\rm{C}}}}_{{{\rm{unfumigated}}}}}{({{{\rm{C}}}}_{{{\rm{fumigated}}}}\,-{{{\rm{C}}}}_{{{\rm{unfumigated}}}})}$$

where MBδ^13^C_fumigated_ is the δ^13^C value of the microbial biomass from the CHCl_3_ fumigated soil-water extract, C_fumigated_ is the size of the C pool size in the CHCl_3_ amended soil-water extract (μg C g^−1^ soil), MBδ^13^C_unfumigated_ is the δ^13^C value of the unamended soil-water extract, and C_unfumigated_ is the C pool size of the unamended soil-water extract (μg C g^−1^ soil). The control and treatment values for MBδ^13^C were then used in Eq. 1 to calculate the fraction of litter-C incorporation into the microbial biomass. The fraction of litter-C incorporation into microbial biomass was multiplied by the microbial biomass pool size (μg MBC g^−1^ soil) to convert to absolute mass of substrate incorporated into the biomass and divided by the original litter addition rate to calculate the% recovery of ^13^C-labelled litter in microbial biomass.

A proxy for microbial CUE was calculated at T15 using the following equation:

3 $${{\rm{CUE}}}=\frac{{{{\rm{MBC}}}}_{{{\rm{litter}}}}}{({{{\rm{MBC}}}}_{{{\rm{litter}}}}+{{{\rm{Respired}}}\; {{\rm{C}}}_{{{\rm{litter}}}}})}$$

where MBC_litter_ is the total litter-C recovered in microbial biomass at T15 and Respired C_litter_ is the cumulative litter-C respired prior to destructive sampling. We did not calculate microbial CUE at T126 as using ^13^C to estimate CUE is sensitive to incubation time and is known to decrease over long term incubations because of microbial turnover^64^. Furthermore, ^13^C tracer can be rapidly lost from biomass and become mineral stabilised as microbial products.

MAOM formation efficiency for each litter and mineral treatment was calculated using the following equation:

4 $${{\rm{Formation\; Efficiency}}}=\frac{{{{\rm{MAOM}}}-{{\rm{C}}}}_{{{\rm{litter}}}}}{({{{\rm{MAOM}}}-{{\rm{C}}}}_{{{\rm{litter}}}}+{{{\rm{Respired}}}\; {{\rm{C}}}_{{\rm{litter}}}})}$$

where MAOM-C_litter_ is the total litter-C recovered in the MAOM fraction and Respired C_litter_ is the litter-C respired throughout the experiment. Formation efficiency is a ratio with a higher value indicative of greater efficiency (more MAOM formed, and less CO_2_ produced per unit of litter processed). Priming effects (PE) were calculated using the following equation:

5 $${{\rm{PE}}}={{{\rm{C}}}}_{{{\rm{som}}}\_{{\rm{treatment}}}}-\,{{{\rm{C}}}}_{{{\rm{som}}}\_{{\rm{control}}}}$$

where C_som_treatment_ is the SOM-derived CO_2_ emissions in litter-amended soils and C_som_control_ is the SOM-derived CO_2_ emissions in the no litter control soils.

### Statistical Analysis

To address H1 that high-quality litter will lead to greater MAOM-C content we used a two-way analysis of variance (ANOVA) with litter-derived MAOM-C and total MAOM-C content as response variables, litter, and mineral treatments as explanatory variables with an interaction term. Post-hoc comparisons between mineral and litter treatments were performed using estimated marginal means using the emmeans (version 1.10.2) R package^109^. To quantify the relative importance of soil mineralogy and litter quality as predictors of MAOM-C content and address H2, a metric of relative importance (lmg) was calculated, partitioning R^2^ and averaging over the order of regressors using the relaimpo (version 2.2–7) R package^110,111^. These metrics are presented as percentages of total explained variance, which sum to the total model R^2^. To investigate the effect of litter chemistry and soil mineralogy on the stabilisation of litter-C in other soil pools (POM and MBC fractions), litter-C loss through microbial respiration (cumulative headspace ^13^CO_2_-C), microbial metrics and priming effects we also used two-way analysis of variance (ANOVA) as described above. Data normality was assessed graphically by plotting Quantile-Quantile (Q-Q) plots and plotting model residuals against fitted values.

To determine whether microbial communities were significantly altered by litter or mineral treatments, we plotted ordinations using non-metric multidimensional scaling (NMDS) on Bray-Curtis dissimilarities and used permutational multivariate analysis of variance (PERMANOVA) to test for the effects of litter and mineral treatments as implemented in the Adonis2 function in the vegan R package (version 2.6-6.1)^112^. To further understand changes in microbial community composition, we calculated the abundance-weighted mean predicted rRNA operon copy number^113^ which has been shown to correlate positively with potential growth rate and inversely with growth efficiency^56,57^. Two- sided Pearson correlations were performed to test the relationship between abundance-weighted mean copy number, relative abundance of fungal soil saprotrophs, litter-C respired and MAOM formation efficiency. All statistics were performed using R version 4.4.1.

### Reporting summary

Further information on research design is available in the Nature Portfolio Reporting Summary linked to this article.

## Supplementary information

Supplementary Information

Reporting Summary

Peer Review File

## Source data

Source Data

## Data Availability

The DNA sequence data generated in this study have been deposited in the European Nucleotide Archive under project accession code PRJEB71146 and are publicly available. The experimental data generated in this study are publicly available at 10.5281/zenodo.13222683. Source data are also provided with this paper. Source data are provided with this paper.

## Code availability

All code is publicly available at 10.5281/zenodo.13222683.
