# Supplementary material for: Microbial and mineral interactions decouple litter quality from soil organic matter formation
Source: Nat Commun. 2024 Nov 20;15:10063. doi: 10.1038/s41467-024-54446-0 (PMC11579368; doi:10.1038/s41467-024-54446-0)

Supplementary information for

Microbial and mineral interactions decouple litter quality from soil organic matter formation

Dafydd M. O. Elias[1], Kelly E. Mason[1], Tim Goodall[2], Ashley Taylor[1], Pengzhi Zhao[1,3], Alba Otero-Fariña[4,5], Hongmei Chen[6], Caroline L. Peacock[4], Nicholas J. Ostle[6], Robert Griffiths[7], Pippa J. Chapman[8], Joseph Holden[8], Steve Banwart[4,9], Niall P. McNamara[1], *Jeanette Whitaker[1]

[1]UK Centre for Ecology & Hydrology, Lancaster Environment Centre, Library Avenue, Bailrigg, Lancaster, United Kingdom, LA1 4AP

[2]UK Centre for Ecology & Hydrology, MacLean Building, Benson Lane, Crowmarsh Gifford, Wallingford, United Kingdom, OX10 8BB

[3]Present Address: Earth and Life Institute, Université Catholique de Louvain, 1348 Louvain-la-Neuve, Belgium

[4]School of Earth and Environment, University of Leeds, Leeds, LS2 9JT, UK

[5]Present Address: CRETUS, University of Santiago de Compostela, 15782, Santiago de Compostela, Spain

[6]Lancaster Environment Centre, Lancaster University, Library Ave, Bailrigg, Lancaster LA1 4YQ, UK

[7]School of Environmental and Natural Sciences, Bangor University, Bangor, Gwynedd, LL57 2DG, UK

[8]water@leeds, School of Geography, University of Leeds, Leeds, LS2 9JT, UK

[9]Global Food and Environment Institute, University of Leeds, Leeds, LS2 9JT, UK

*Corresponding Author: Jeanette Whitaker jhart@ceh.ac.uk

This file contains:

Supplementary tables 1-8 Supplementary figures 1-6

Supplementary Tables

Supplementary Table 1: Two-way ANOVA results for the effects of soil mineralogy (Mineral) and litter quality (Litter) and an interaction term on the proportion of litter-derived carbon (C) respired and primed soil organic matter (SOM) after 126 days of incubation (T126). Litter-derived C respired was square-root transformed for normality. Relative importance was calculated by averaging over orders of regressors and is presented as percentages which sum to the overall model R^2.

	Numerator df	Cumulative litter-derived CO_2-C			Primed SOM-derived CO_2-C		
Timepoint		T126			T126		
Units		%	%		µg CO_2-C g^{-1} dry soil	%	
		F (P)	Relative Importance	Model R^2	F (P)	Relative Importance	Model R^2
Mineral	3	**268.66** (<2.20E-16)	57.2	97.7	**7.27** (7.49E-04)	22.2	67.4
Litter	1	**399.43** (<2.20E-16)	28.3		**29.27** (6.02E-06)	29.8	
Mineral x Litter	3	**57.41** (5.63E-13)	12.2		**5.02** (5.77E-03)	15.3	

Supplementary Table 2: Two-way ANOVA results for the effects of soil mineralogy (Mineral) and litter quality (Litter) and an interaction term on the proportion of litter-derived carbon (C) recovered in particulate organic matter (POM), mineral-associated organic matter (MAOM) and sand after 126 days of incubation (T126). POM was log-transformed for normality. Relative importance was calculated by averaging over orders of regressors and is presented as percentages which sum to the overall model R^2.

	Numerator df	MAOM (>1.8g cm $^{-3}$, <53μm)			Sand (>1.8 g cm $^{-3}$, >53μm)			POM		
Timepoint		T126			T126			T126		
Units		%	%		%	%		%	%	
		F (P)	Relative Importance	Model R^2	F (P)	Relative Importance	Model R^2	F (P)	Relative Importance	Model R^2
Mineral	3	**44.35** (1.66E-11)	51.4	87.7	**3.92** (1.73E-02)	12.4	66.3	**32.93** (6.68E-10)	49.7	83.9
Litter	1	**53.94** (2.37E-08)	20.8		**47.31** (8.78E-08)	49.8		**66.55** (2.57E-09)	33.5	
Mineral x Litter	3	**13.34** (8.12E-08)	15.5		1.30 (0.29)	4.1		0.45 (0.72)	0.7	

Supplementary Table 3: Two-way ANOVA results for the effects of soil mineralogy (Mineral) and litter quality (Litter) and an interaction term on the proportion of litter-derived carbon (C) recovered in microbial biomass (MBC) after 15 days (T15) and 126 days (T126) of incubation. MBC at T15 was square root transformed for normality. Relative importance was calculated by averaging over orders of regressors and is presented as percentages which sum to the overall model R^2.

	Numerator df	MBC			MBC		
Timepoint		T15			T126		
Units		%	%		%	%	
		F (P)	Relative Importance	Model R^2	F (P)	Relative Importance	Model R^2
Mineral	3	**5.12** (5.76E-03)	25.8	54.1	1.27 (0.30)	9.4	21.4
Litter	1	**8.97** (5.58E-03)	15.7		0.80 (0.38)	2.0	
Mineral x Litter	3	2.67 (0.07)	12.6		1.36 (0.27)	10.0	

Supplementary Table 4: Two-way ANOVA results for the effects of soil mineralogy (Mineral) and litter quality (Litter) and an interaction term on the mineral-associated organic matter (MAOM) formation efficiency (FE) and total MAOM stocks. FE was square root transformed for normality. Relative importance was calculated by averaging over orders of regressors and is presented as percentages which sum to the overall model R^2.

	**Numerator df**	**FE**			**Total MAOM Stocks**		
Timepoint		T126			T126		
Units		Unitless	%	%	mg C g^{-1} dry soil	%	%
		F (P)	Relative Importance	Model R^2	F (P)	Relative Importance	Model R^2
Mineral	3	**168.32** (<2.20E-16)	57.3	96.4	**42.66** (2.25E-11)	18.1	72.3
Litter	1	**221.78** (6.09E-16)	25.2		**10.45** (2.09E-05)	49.3	
Mineral x Litter	3	**40.96** (4.57E-11)	13.9		1.40 (0.23)	4.9	

Supplementary Table 5: Two-way ANOVA results for the effects of soil mineralogy (Mineral) and litter quality (Litter) and an interaction term on microbial biomass carbon concentrations (MBC), bacterial and fungal richness after 15 (T15) and 126 days (T126) of incubation. MBC at T15 and T126 were log transformed for normality. Relative importance was calculated by averaging over orders of regressors and is presented as percentages which sum to the overall model R^2.

	Numerator df	MBC			Bacterial Richness			Fungal Richness		
Units		µg C g^{-1} dry soil	%	%	integer	%	%	Integer	%	%
**T15**		F (P)	Relative Importance	Model R^2	F (P)	Relative Importance	Model R^2	F (P)	Relative Importance	Model R^2
Mineral	3	0.91 (0.44)	1.5	88.9	0.51 (0.68)	2.7	16.3	2.17 (0.10)	2.3	82.1
Litter	2	**166.29** (<2E-16)	84.7		1.87 (0.17)	6.7		**96.22** (<2.00E-16)	76.4	
Mineral x Litter	6	1.80 (0.12)	2.7		0.63 (0.70)	6.9		1.42 (0.22)	3.4	
**T126**										
Mineral	3	0.79 (0.50)	1.9	62.1	0.51 (0.68)	2.9	8.4	1.65 (0.19)	1.6	87.6
Litter	2	**34.43** (5.31E-10)	54.4		0.59 (0.56)	2.2		**142.33** (2.20E-16)	79.7	
Mineral x Litter	6	1.23 (0.31)	5.8		0.29 (0.94)	3.3		**3.78** (4.03E-03)	6.3	

Supplementary Table 6: PERMANOVA and two-way ANOVA results for the effects of soil mineralogy (Mineral) and litter quality (Litter) and an interaction term on bacterial community composition and abundance-weighted rRNA operon copy number. Minimum p values for permutation tests = 1/(N+1), where N is the number of permutations. PERMANOVA P values were generated with 1E6 permutations with a minimum p-value of 1.00E-06. Relative importance was calculated by averaging over orders of regressors and is presented as percentages which sum to the overall model R^2.

	Numerator df	Bacterial Communities						Bacterial rRNA operon copy number					
Timepoint		T15			T126			T15			T126		
Units		Unit interval	%	%	Unit interval	%	%	Integer	%	%	Integer	%	%
		F (P)	Relative Importance	Model R^2	F (P)	Relative Importance	Model R^2	F (P)	Relative Importance	Model R^2	F (P)	Relative Importance	Model R^2
Mineral	3	**3.46** (2.00E-06)	10.7	52.5	**3.83** (1.00E-06)	13	45.6	**10.67** (1.82E-05)	10.7	84.6	**25.65** (4.76E-10)	25.8	84.0
Litter	2	**15.20** (1.00E-06)	31.3		**9.15** (1.00E-06)	20.7		**98.13** (<2.2E-16)	64.3		**53.04** (6.97E-13)	35.6	
Mineral x Litter	6	**1.70** (1.93E-03)	10.5		**1.75** (1.70E-05)	11.9		**4.92** (5.58E-04)	9.6		**11.23** (8.52E-08)	22.6	

Supplementary Table 7: PERMANOVA and two-way ANOVA results for the effects of soil mineralogy (Mineral) and litter quality (Litter) and an interaction term on fungal community composition and the relative abundance of soil and litter saprotrophs. Minimum p values for permutation tests = 1/(N+1), where N is the number of permutations. PERMANOVA P values were generated with 1E6 permutations with a minimum p-value of 1.00E-06. Relative importance was calculated by averaging over orders of regressors and is presented as percentages which sum to the overall model R^2.

	Numerator df	Fungal Communities			Fungal Soil Saprotrophic Abundance			Fungal Litter Saprotrophic Abundance		
Units		Unit interval	%	%	Unit interval	%	%	Unit interval	%	%
**T15**		F (P)	Relative Importance	R^2	F (P)	Relative Importance	R^2	F (P)	Relative Importance	R^2
Mineral	3	**4.85** (5.00E-06)	8.8	72.7	0.18 (0.91)	0.5	66.5	**16.36** (2.48E-07)	27	76.3
Litter	2	**42.71** (1.00E-06)	51.9		**28.98** (8.16E-09)	43.2		**41.63** (5.82E-11)	45	
Mineral x Litter	6	**3.29** (1.10E-05)	12		**5.11** (4.47E-04)	22.8		1.36 (0.25)	4.3	
**T126**										
Mineral	3	**5.74** (1.00E-06)	13.2	66.3	**6.87** (6.81E-04)	12.4	73.3	**4.33** (9.32E-03)	10.5	65.0
Litter	2	**27.84** (1.00E-06)	42.6		**42.74** (4.87E-11)	51.7		**31.86** (2.79E-09)	50.9	
Mineral x Litter	6	**2.28** (4.70E-04)	10.5		**2.54** (0.03)	9.2		0.75 (0.61)	3.6	

Supplementary Table 8: MAOM carbon (C) and nitrogen (N) concentrations and C:N ratios measured from microcosms after 126 days of incubation (T126). Data are means (*n* = 5 in each treatment) with the standard error in parentheses.

Timepoint	Mineral	Litter	MAOM-C [%]	MAOM-N [%]	MAOM C:N
T126	No Minerals	No Litter	5.05 (0.06)	0.51 (0.01)	9.87 (0.06)
T126	Kaolinite	No Litter	4.00 (0.16)	0.40 (0.01)	9.91 (0.11)
T126	Goethite	No Litter	3.86 (0.06)	0.41 (0.01)	9.48 (0.08)
T126	Montmorillonite	No Litter	4.00 (0.09)	0.41 (0.01)	9.67 (0.07)
T126	No Minerals	Low Quality	4.98 (0.10)	0.51 (0.01)	9.82 (0.14)
T126	Kaolinite	Low Quality	4.06 (0.04)	0.40 (0.01)	10.07 (0.14)
T126	Goethite	Low Quality	3.91 (0.06)	0.39 (0.01)	9.97 (0.22)
T126	Montmorillonite	Low Quality	3.40 (0.07)	0.34 (0.01)	9.90 (0.26)
T126	No Minerals	High Quality	5.01 (0.13)	0.52 (0.01)	9.60 (0.11)
T126	Kaolinite	High Quality	3.66 (0.08)	0.39 (0.01)	9.33 (0.22)
T126	Goethite	High Quality	3.84 (0.08)	0.41 (0.01)	9.34 (0.27)
T126	Montmorillonite	High Quality	3.59 (0.09)	0.38 (0.01)	9.41 (0.08)

Supplementary Figures

Supplementary Figure 1: Effects of litter quality and soil mineralogy on soil bacterial communities at early-stage decomposition and relationship to litter carbon (C) stabilisation and loss. Soil bacterial community composition at early-stage decomposition (15 Days: T15) (**a**), abundance weighted mean rRNA gene copy number of bacterial communities at T15 (**b**) and relationships between the abundance weighted mean rRNA gene copy number of bacterial communities and the percentage of litter-derived C respired up to T15 (**c**). 1 sample was excluded (Kaolinite: High Quality) due to sample loss and 1 sample was excluded due to a low number of sequence reads (No Minerals: No Litter). Bacterial communities are presented using non-metric multidimensional scaling analysis conducted on a Bray-Curtis dissimilarity matrix demonstrating strong impacts of the litter (labelled and colour-coded spiders) and mineral (indicated by symbol shape) treatments. In panel b, bars indicate data means (*n* = 5) ± 1 standard error (displayed as error bars) (*n* = 4 for Kaolinite: High Quality and No Minerals: No Litter treatments). Raw data are overlaid on bar charts to show the underlying data distribution. Statistics were derived from *n* = 5 (*n* = 4 for treatments described above) independent samples. Between group differences in abundance-weighted mean 16S rRNA gene copy number were compared using estimated marginal means (emmeans) to determine the effect of litter quality by mineral treatment. The two-sided p-values from emmeans tests were adjusted for multiple comparisons using the Benjamini–Hochberg (BH) procedure and presented within the plots. Statistics in panel c and d were derived from *n* = 5 independent samples (*n* = 4 for Kaolinite: High Quality). Solid lines represent two-sided pearson correlations at $p < 0.05$. P values derived from correlations were not adjusted for multiple comparisons. The sample size '*n*' represents samples taken from independent experimental units (soil incubations). Source data are provided as a Source Data file. Exact P values are available in the corresponding Source Data file.

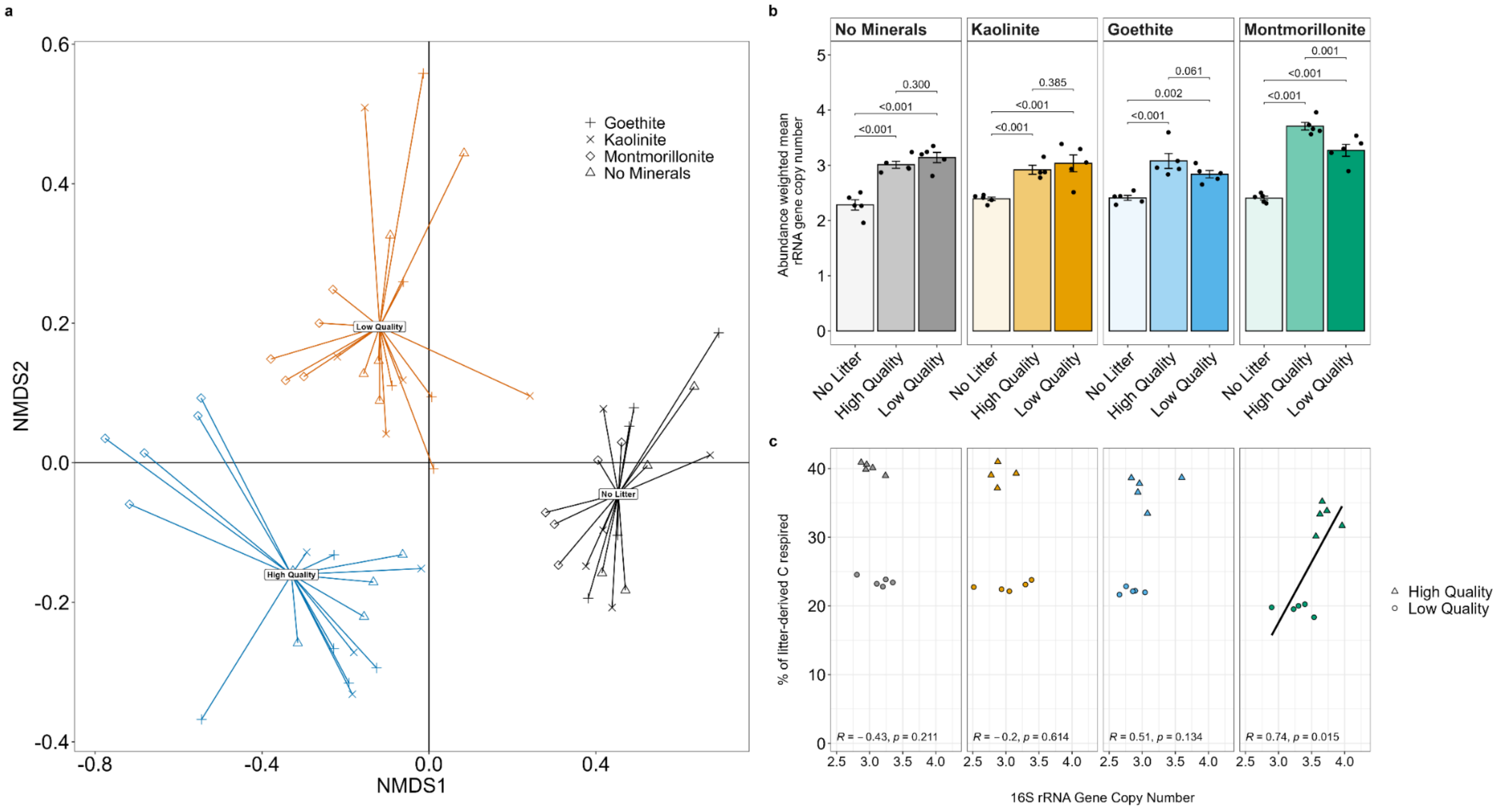
a
b
c
NMDS1
NMDS2
Goethite
Kaolinite
Montmorillonite
No Minerals
Low Quality
High Quality
No Litter
No Minerals
Kaolinite
Goethite
Montmorillonite
Abundance weighted mean rRNA gene copy number
<0.001
0.300
0.385
0.061
0.002
0.001
No Litter
High Quality
Low Quality
% of litter-derived C respired
R = −0.43, p = 0.211
R = −0.2, p = 0.614
R = 0.51, p = 0.134
R = 0.74, p = 0.015
16S rRNA Gene Copy Number
High Quality
Low Quality

Supplementary Figure 2: **Effects of litter quality and soil mineralogy on soil fungal communities at early-stage decomposition and relationship to litter carbon (C) stabilisation and loss.** Relative abundance of fungal saprotrophs partitioned into specialist litter and generalist soil saprotrophs at early-stage decomposition (15 days: T15) (**a**), soil fungal community composition at T15 (**b**), relationship between soil saprotrophs and percentage of litter-derived C respired at T15 (**c**) and the relationship between litter saprotrophs and percentage of litter-derived C respired at T15 (**d**). 1 sample was excluded (Kaolinite: High Quality) due to sample loss and 2 samples were excluded due to a low number of sequence reads (No Minerals: No Litter; Montmorillonite: Low Quality). Fungal communities in panel a are presented using non-metric multidimensional scaling analysis conducted on a Bray-Curtis dissimilarity matrix demonstrating strong impacts of the litter (labelled and colour-coded spiders) and mineral (indicated by symbol shape) treatments. In panel b, bars indicate data means (n = 5) ± 1 standard error (displayed as error bars) (n = 4 for Kaolinite: High Quality, No Minerals: No Litter and Montmorillonite: Low Quality treatments). Raw data are overlaid on bar charts to show the underlying data distribution. Statistics in panel c and d were derived from n = 5 independent samples (n = 4 for Kaolinite: High Quality and Montmorillonite: Low Quality treatments). Solid lines represent two-sided pearson correlations at $p < 0.05$. P values derived from correlations were not adjusted for multiple comparisons. The sample size 'n' represents samples taken from independent experimental units (soil incubations). Source data are provided as a Source Data file. Exact P values are available in the corresponding Source Data file.

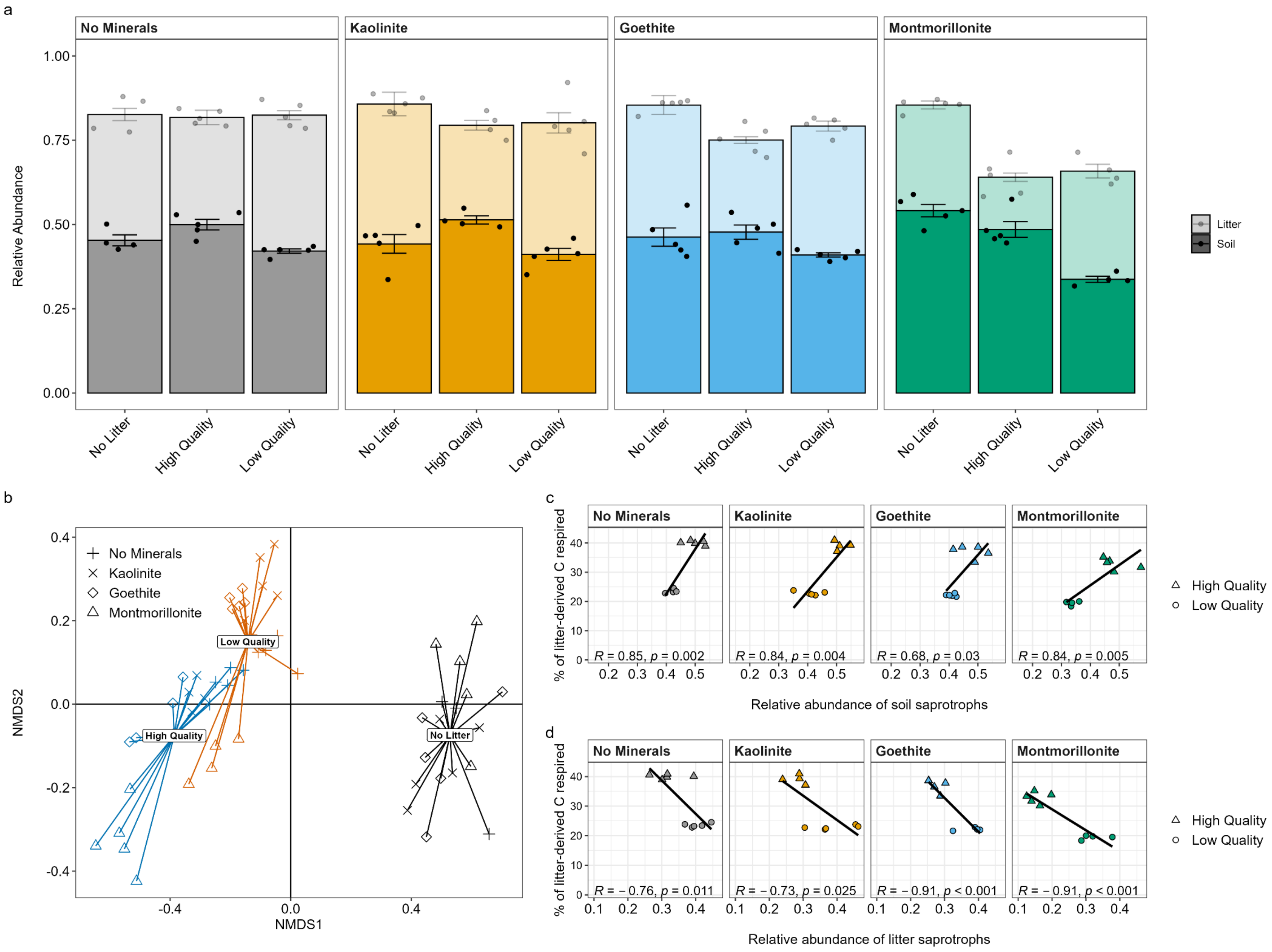
a
No Minerals
Kaolinite
Goethite
Montmorillonite
Relative Abundance
1.00
0.75
0.50
0.25
0.00
No Litter
High Quality
Low Quality
Litter
Soil
b
No Minerals
Kaolinite
Goethite
Montmorillonite
Low Quality
High Quality
No Litter
NMDS2
NMDS1
0.4
0.2
0.0
-0.2
-0.4
c
% of litter-derived C respired
R = 0.85, p = 0.002
R = 0.84, p = 0.004
R = 0.68, p = 0.03
R = 0.84, p = 0.005
Relative abundance of soil saprotrophs
High Quality
Low Quality
d
R = −0.76, p = 0.011
R = −0.73, p = 0.025
R = −0.91, p < 0.001
R = −0.91, p < 0.001
Relative abundance of litter saprotrophs

Supplementary Figure 3: The relative abundance of litter saprotrophs determines the amount of carbon (C) respired from added litter across soils. Relationship between the relative abundance of litter saprotrophs and the percentage of litter-derived C respired at early-stage decomposition (T15). 1 sample (Kaolinite: High Quality) was excluded due to sample loss. 1 sample (Montmorillonite: Low Quality) was excluded due to a low number of sequence reads. All statistics were derived from *n* = 19 independent samples. Solid black lines indicate two-sided pearson correlations at $p < 0.05$. P values derived from correlations were not adjusted for multiple comparisons. The sample size '*n*' represents samples taken from independent experimental units (soil incubations). Source data are provided as a Source Data file. Exact P values are available in the corresponding Source Data file.

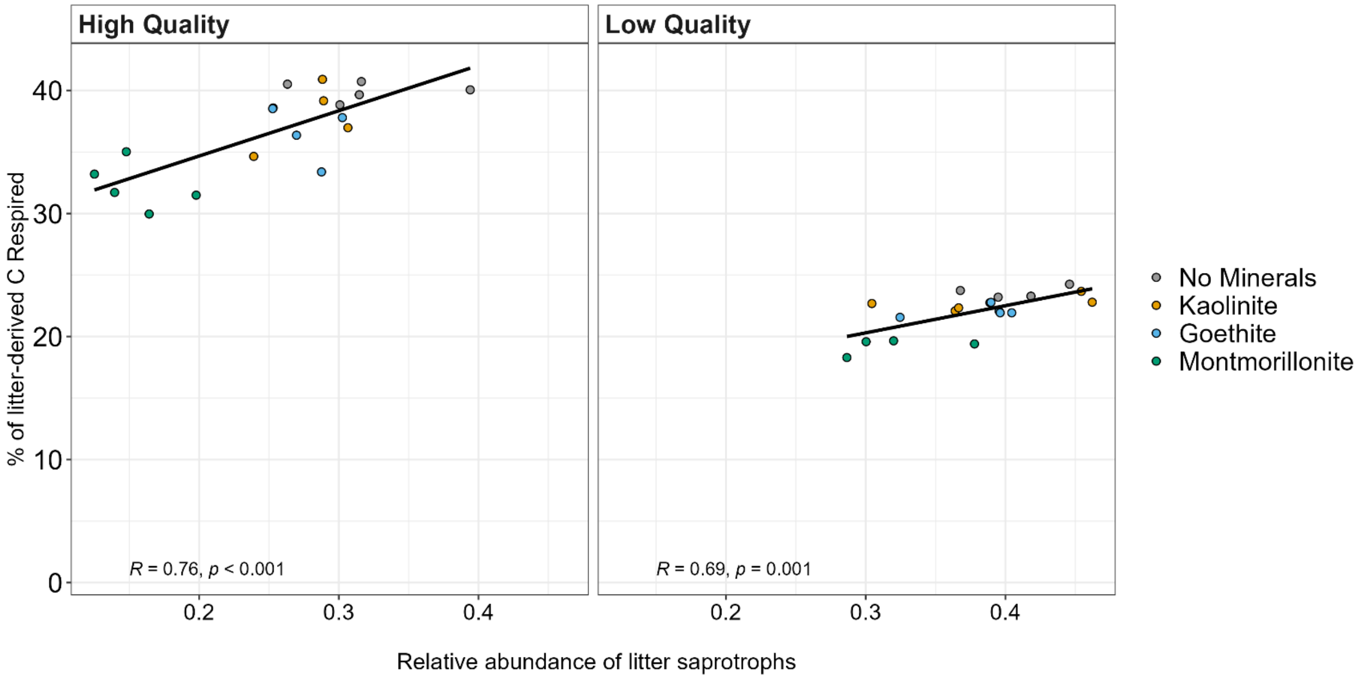

Supplementary Figure 4: Effects of litter quality and soil mineralogy on the relative abundance of soil saprotrophic fungal genera at early- and late-stage decomposition. Relative abundance of soil saprotrophic fungal genera at early-stage (15 days: T15) decomposition (**a**) and late-stage (126 days: T126) decomposition (**b**). All individual samples are displayed. 1 sample was excluded at timepoint T15 due to sample loss. 2 and 4 samples were excluded from T15 and T126 respectively due to a low number of sequence reads. Fungal taxa were assigned as soil saprotrophic using the FungalTraits database. Low abundance genera (<5 % relative abundance) were grouped and assigned as '<5% Abundance'. Source data are provided as a Source Data file.

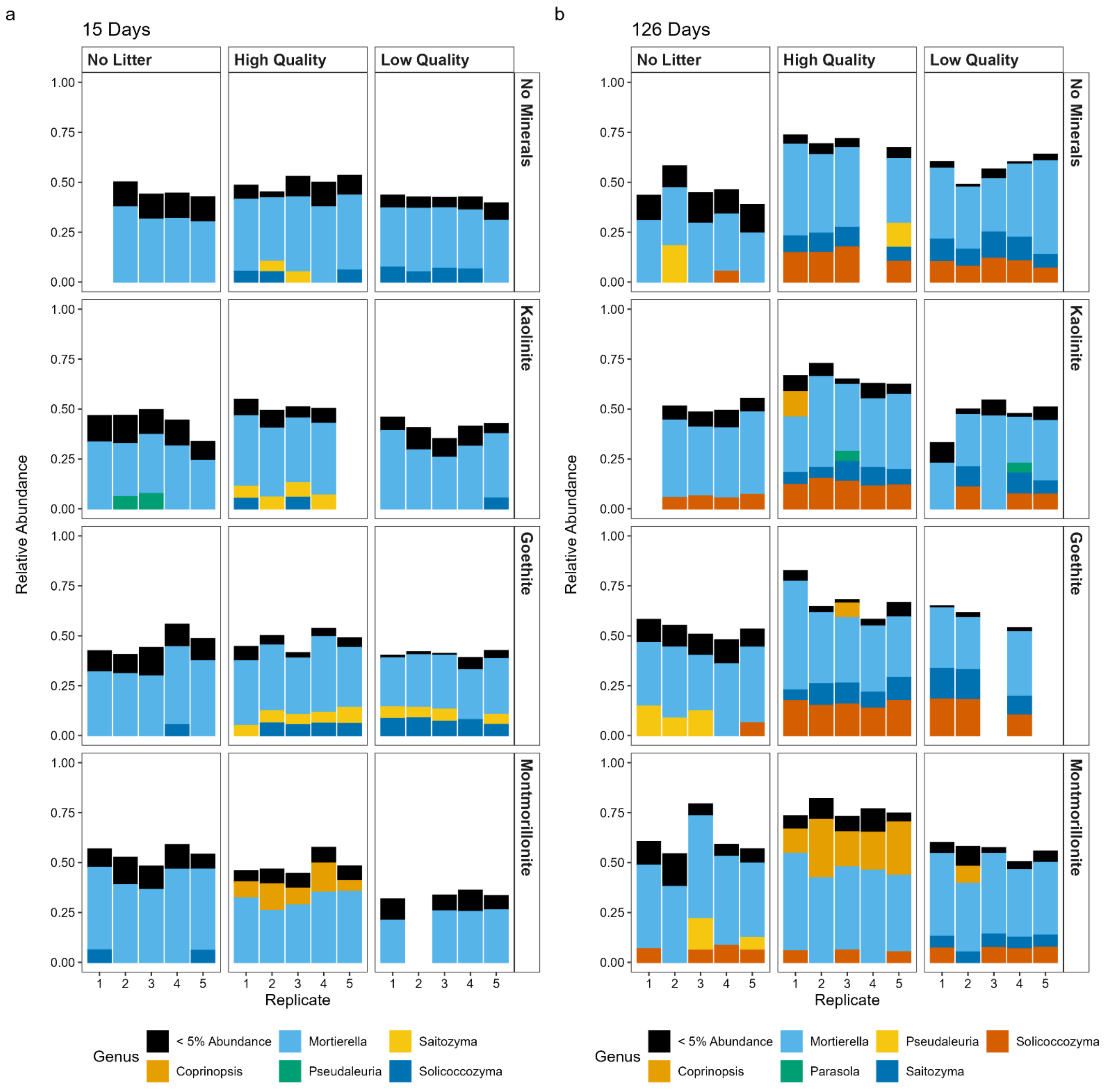

Supplementary Figure 5: Effects of litter quality and soil mineralogy on the relative abundance of litter saprotrophic fungal genera at early- and late-stage decomposition. Relative abundance of litter saprotrophic fungal genera at early-stage (15 days: T15) decomposition (**a**) and late-stage (126 days: T126) decomposition (**b**). All individual samples are displayed. 1 sample was excluded at timepoint T15 due to sample loss. 2 and 4 samples were excluded from T15 and T126 respectively due to a low number of sequence reads. Fungal taxa were assigned as litter saprotrophic using the FungalTraits database. Low abundance genera (<5 % relative abundance) were grouped and assigned as '<5% Abundance'. Source data are provided as a Source Data file.

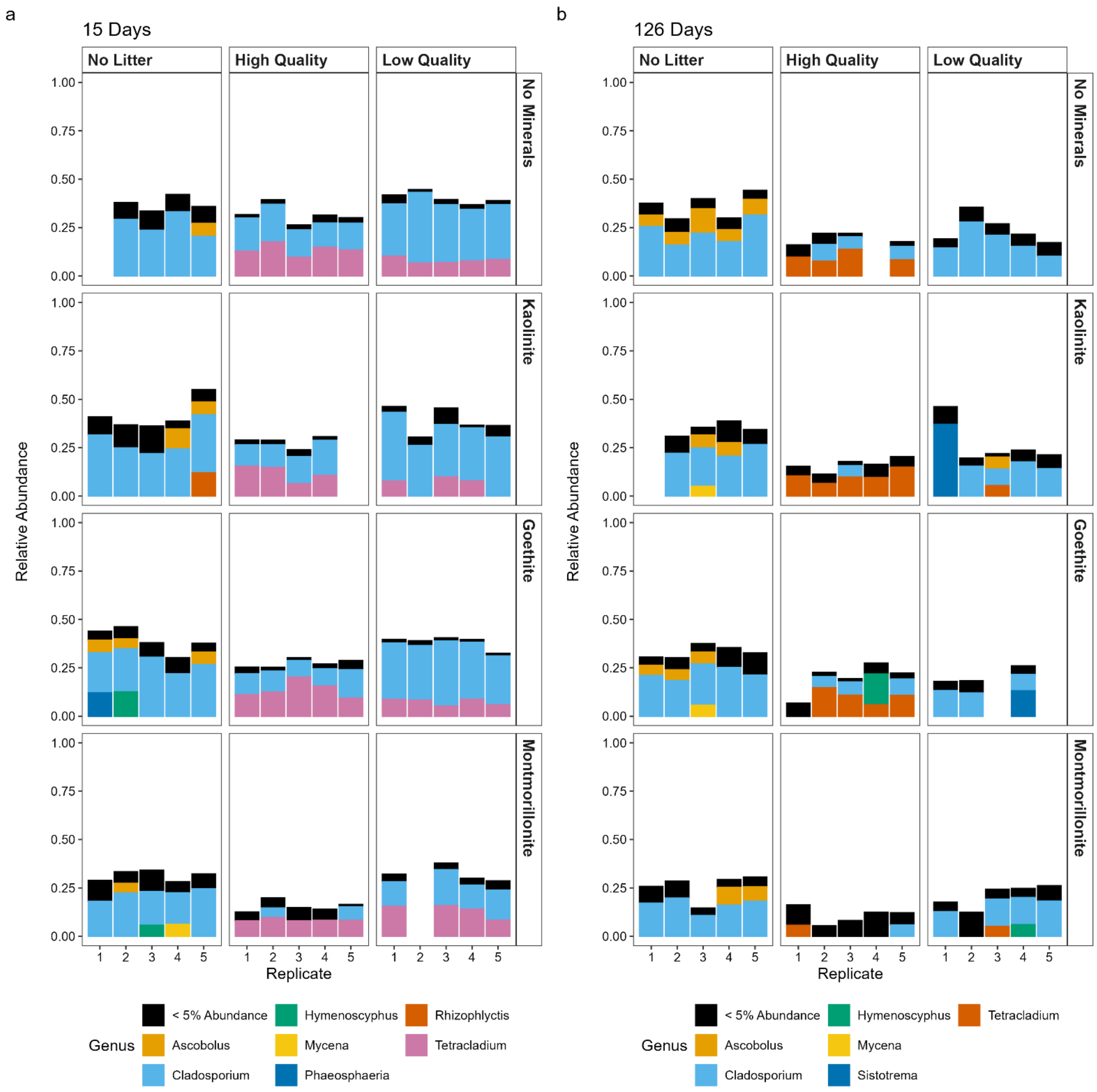

Supplementary Figure 6: Schematic of the soil fractionation procedure used to separate particulate organic matter (POM) and mineral-associated organic matter (MAOM). Key: POM_{free} = non-occluded particulate organic matter with density < 1.8 g cm^{-3}, $POM_{occluded}$ = particulate organic matter occluded within aggregates, POM = particulate organic matter free and within aggregates with density < 1.8 g cm^{-3}, Sand = particles >53µm in size and with density > 1.8 g cm^{-3}, MAOM = mineral-associated organic matter defined as particles <53µm and with density > 1.8 g cm^{-3}.

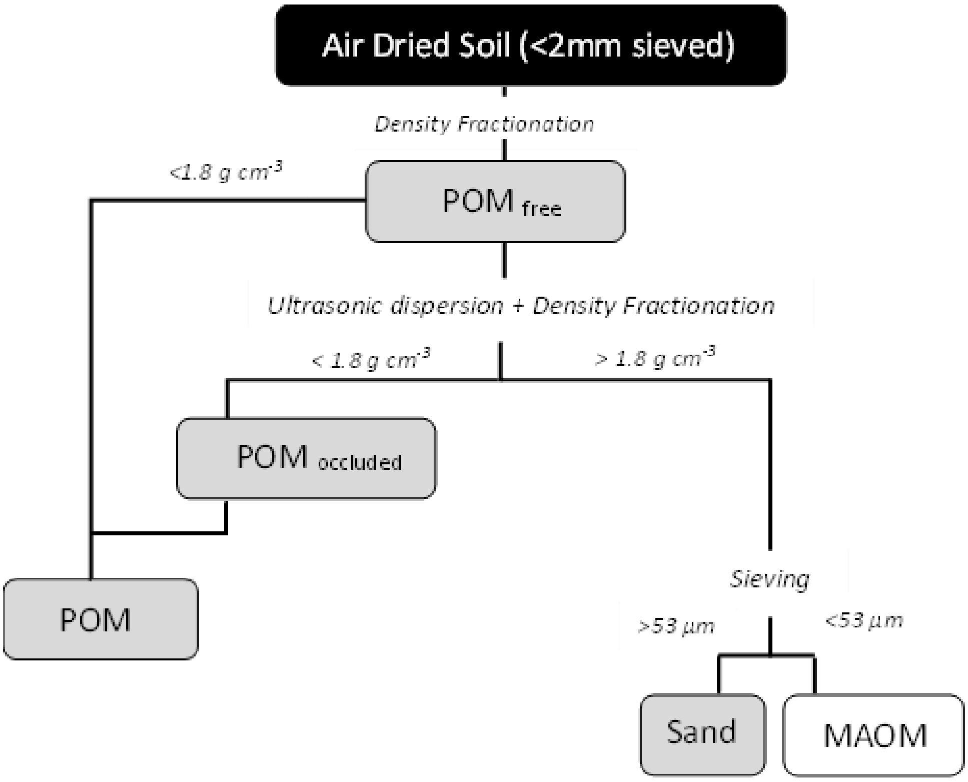

Supplement: Supplementary file 1 — Supplementary Information [file 41467_2024_54446_MOESM1_ESM.pdf]
