# Supplementary material for: Microbial and mineral interactions decouple litter quality from soil organic matter formation
Source: Nat Commun. 2024 Nov 20;15:10063. doi: 10.1038/s41467-024-54446-0 (PMC11579368; doi:10.1038/s41467-024-54446-0)
Supplement: Supplementary file 2 — Reporting Summary [file 41467_2024_54446_MOESM2_ESM.pdf]

nature portfolio

Corresponding author(s): Jeanette Whitaker

Last updated by author(s): 30/10/2024

Reporting Summary

Nature Portfolio wishes to improve the reproducibility of the work that we publish. This form provides structure for consistency and transparency in reporting. For further information on Nature Portfolio policies, see our Editorial Policies and the Editorial Policy Checklist.

Statistics

For all statistical analyses, confirm that the following items are present in the figure legend, table legend, main text, or Methods section.

n/a	Confirmed	
☐	☒	The exact sample size (*n*) for each experimental group/condition, given as a discrete number and unit of measurement
☐	☒	A statement on whether measurements were taken from distinct samples or whether the same sample was measured repeatedly
☐	☒	The statistical test(s) used AND whether they are one- or two-sided<br>*Only common tests should be described solely by name; describe more complex techniques in the Methods section.*
☐	☒	A description of all covariates tested
☐	☒	A description of any assumptions or corrections, such as tests of normality and adjustment for multiple comparisons
☐	☒	A full description of the statistical parameters including central tendency (e.g. means) or other basic estimates (e.g. regression coefficient) AND variation (e.g. standard deviation) or associated estimates of uncertainty (e.g. confidence intervals)
☐	☒	For null hypothesis testing, the test statistic (e.g. F, t, r) with confidence intervals, effect sizes, degrees of freedom and P value noted<br>*Give P values as exact values whenever suitable.*
☒	☐	For Bayesian analysis, information on the choice of priors and Markov chain Monte Carlo settings
☒	☐	For hierarchical and complex designs, identification of the appropriate level for tests and full reporting of outcomes
☐	☒	Estimates of effect sizes (e.g. Cohen's *d*, Pearson's *r*), indicating how they were calculated

Our web collection on statistics for biologists contains articles on many of the points above.

Software and code

Policy information about availability of computer code

Data collection	No software was used to collect data
Data analysis	The R package "dada2" version 1.28.0 was used for processing of Illumina demultiplexed raw sample data using R version 4.3.1.<br>The phyloseq R package (version 1.48.0) was used to visualise and filter microbial data using R version 4.4.1. All statistical analyses were conducted using R version 4.4.1 in the RStudio IDE version 2024.09.0 Build 375. Post-hoc comparisons between treatments were performed using the R package "Emmeans" (version 1.10.2). To quantify the relative importance of predictors the "Relaimpo" (version 2.2-7) R package was used. To determine whether microbial community composition differed between treatments the Vegan R package (version 2.6-6.1) was used.

For manuscripts utilizing custom algorithms or software that are central to the research but not yet described in published literature, software must be made available to editors and reviewers. We strongly encourage code deposition in a community repository (e.g. GitHub). See the Nature Portfolio guidelines for submitting code & software for further information.

Data

Policy information about availability of data

All manuscripts must include a data availability statement. This statement should provide the following information, where applicable:

- Accession codes, unique identifiers, or web links for publicly available datasets
- A description of any restrictions on data availability
- For clinical datasets or third party data, please ensure that the statement adheres to our policy

Data Availability
The DNA sequence data generated in this study have been deposited in the European Nucleotide Archive under project accession code PRJEB71146 and are publicly available. The experimental data generated in this study are publicly available at https://doi.org/10.5281/zenodo.13222683. Source data are also provided with this paper.

Code Availability
All code is publicly available at https://doi.org/10.5281/zenodo.13222683

Research involving human participants, their data, or biological material

Policy information about studies with human participants or human data. See also policy information about sex, gender (identity/presentation), and sexual orientation and race, ethnicity and racism.

Reporting on sex and gender	No human participants involved
Reporting on race, ethnicity, or other socially relevant groupings	No human participants involved
Population characteristics	No human participants involved
Recruitment	No human participants involved
Ethics oversight	Not relevant

Note that full information on the approval of the study protocol must also be provided in the manuscript.

Field-specific reporting

Please select the one below that is the best fit for your research. If you are not sure, read the appropriate sections before making your selection.

☐ Life sciences ☐ Behavioural & social sciences ☒ Ecological, evolutionary & environmental sciences

For a reference copy of the document with all sections, see nature.com/documents/nr-reporting-summary-flat.pdf

Ecological, evolutionary & environmental sciences study design

All studies must disclose on these points even when the disclosure is negative.

Study description	We investigated the effect of litter quality on the formation of mineral associated organic matter across soils of contrasting mineralogies. The study included a 126 day incubation, where isotopically labelled litter was added to soils amended with pristine minerals to determine how litter quality influenced the formation and mineralization of new and native soil organic matter (SOM). Soil microbial communities were characterized at early and late-stages of litter decomposition to investigate how microbial taxonomy and physiology linked litter quality to SOM stabilization.<br><br>The study was designed as a 4x3 full factorial design conducted in the laboratory under controlled environmental conditions. The experimental units were soil incubations (soil in 1L kilner jars) with soil mineralogy and litter quality as treatment factors with an interaction term. Soil mineralogy is a 4 level factor (No minerals, Kaolinite, Goethite, Montmorillonite), Litter quality is a 3 level factor (No litter, low quality, high quality) with 5 replicates of each for 60 experimental units. The experiment was duplicated to allow for destructive sampling after 15 days (referred to as T15 in the manuscript) and 126 days (T126) for a total of 10 replicates and 120 experimental units. The 5 replicate experimental units destructively harvested after 15 days were used to assess the short-term microbial response to litter addition while the remaining 5 replicate microcosms were used for the bulk quantification of respired litter-C and transfer to SOM fractions after 126 days of incubation.
Research sample	The soil used in this study was sampled from an agricultural field near Glassonby, Penrith. This sandy loam soil was chosen due to its coarse texture (sandy loam), which was suitable for amendment with pristine minerals. These soils are also widespread across the UK and commonly used for agriculture. The litter material used in this study was grown in house and produced from winter wheat (low quality) and white clover (high quality). These two plant litters were chosen as they differ strongly in chemical quality, have widely

differing C:N ratios and are both commonly grown in the UK as a cereal, cover crop or understorey.

Sampling strategy

To account for within field variation of soil, we collected 40 soil cores (15cm depth) across the field in a W sampling pattern. No pre-calculation of sample-size was performed as the objective was to collect field representative soil in a sufficient quantity rather than characterize within-field variability. In the lab root material and rocks were removed and soils were passed through a 4mm sieve. This soil homogenization was performed to reduce variability between experimental units (microcosms). The plant litter was dried and screened through a 250 micron sieve and added individually to soil microcosms assigned to each litter treatment. The incubation was carried out on 10 replicates per treatment of which 5 were destructively harvested after 15 days and 5 after 126 days.

Sampling to capture CO2 respiration in incubation jars was conducted throughout the duration of the experiment from 5 replicates to capture cumulative respired loss of litter carbon. CO2 data was collected daily from experimental units at days 0-10 and then on days 13, 15, 17, 20, 24, 28, 31, 34, 38, 41, 45, 49, 56, 63, 70, 77, 84, 91, 98, 105, 112, 119 and 126. This was designed to capture initial high microbial activity rates following litter addition.

Microbial metrics were measured on soils at destructive harvests that took place after 15 and 126 days to quantify microbial biomass and community dynamics at two differing stages of litter decomposition (early and late). Soil fractions were sampled and quantified after 126 days only to address our primary hypothesis regarding the effect of litter quality on the formation of new mineral associated organic matter.

5 replicates was chosen as the level of replication (per timepoint) based on a balance of statistical power and practical considerations of the sampling effort required to run the laboratory experiment. No formal sample-size calculation was performed.

Data collection

DE and KM carried out and performed the soil CO2 respiration measurements using manual syringe extraction, injection into evacuated glass vials and analysis on a Gas Chromatograph. DE, KM, AT, HC, PZ carried out microbial biomass fumigation extractions and soil density fractionation. Organic carbon and d13c in liquid samples was determined by DE and KM using an Aurora 1030W TOC analyzer coupled to a Picarro G2201i CRDS analyzer. Solid samples were analyzed for organic carbon using a LECO Truspec elemental analyzer by DE and KM and d13c was determined using a Costech ECS4010 elemental analyzer coupled to a Picarro G2131i CRDS analyzer. DNA extraction and rRNA (16S, ITS) sequencing was conducted by TG. DNA was extracted from 0.2 g frozen soil using a Powersoil® DNA Isolation Kit according to the manufacturer's instructions. Bacterial and fungal community compositions were assessed by sequencing the V4-V5 region of the 16S rRNA genes using the 515f GTGYCAGCMGCCGCGGTAA and 806r GGACTACNVGGGTWTCTAAT primers 102 and the established primers GTGARTCATCGAATCTTTG and TCCTCCGCTTATTGATATGC coding the ITS2 region 100. Each amplicon library was sequenced separately using a 2-step Nextera approach on the Illumina MiSeq platform with V2 500 cycle chemistry (Illumina Inc., USA), with 8 pM loads and 7.5% phiX control library. Raw sequencing data is available at the European Nucleotide Archive under project accession code PRJEB71146

Timing and spatial scale

The incubation experiment was run for 126 days between 10/2020 - 03/2021. CO2 respiration data was collected daily from experimental units at days 0-10 and then on days 13, 15, 17, 20, 24, 28, 31, 34, 38, 41, 45, 49, 56, 63, 70, 77, 84, 91, 98, 105, 112, 119 and 126. The decreasing frequency of respiration measurements was designed to capture initial high microbial activity rates following litter addition which typically decline over time. The incubation length was chosen to allow time for most of the litter to be processed by microbes and transformed from particulate matter (Litter-C remaining as POM varied from 0.2 - 6.9 %). CO2 respiration rates and respiration of litter-derived C was monitored during the experiment and microcosms were destructively harvested once litter-C mineralization appeared to stabilize. Soils data was collected by destructive harvesting of replicate experimental units (5 reps) on day 15 and day 126 to capture differing stages of litter decomposition. Microbial biomass extractions were performed in 10/2020 and 03/2021 immediately after destructive harvests. Soil density fractionation was performed between 10/2021-12/2021. DNA extraction and sequencing was done in 02/2022. Bulk analytical data is taken from cm scale.

Data exclusions

1 experimental unit was excluded at timepoint T15 due to sample loss from damage to the incubation jar. Data from 3 microbial biomass extractions at T15 were excluded due to poor extraction/sample degradation. 1 16S bacterial and 6 ITS fungal community samples were excluded from analysis due to a low number of sequence reads.

Reproducibility

Pilot testing was performed on soil incubations to determine the optimal amount of soil and litter, moisture conditions and volume of incubation jar to achieve optimal microbial activity and soil CO2 respiration rates that would be easily detectable but not excessive such that headspaces would quickly become depleted in oxygen within the sampling timings planned (See timing and spatial scale above). We found that 75g of soil at 65% of maximum water holding capacity with 1g of litter in a 1L incubation jar produced adequate CO2 respiration rates and provided sufficient soil for analyses planned. We also pilot tested the energy required to disperse soils during density fractionation to minimise the redistribution of C across fractions. The lowest energy required to fully disperse soils was determined by observing the effects of a stepwise increase of sonication energy on liberated light material. Sonication energy was applied in 50 J ml-1 increments up to 300 J ml-1 and floating light material removed by pipetting prior to each subsequent sonication of 50 J ml-1. Complete disruption of aggregates was assumed when no further material was observed floating in the SPT solution. We found that soils were weakly aggregated as 100 J ml-1 was sufficient to fully disperse our soils. The experimental results show high reproducibility between replicates. The pilot soil incubations and fractionation pilot were performed once before the main experiment. However, no attempt was made to repeat the entire experiment.

Randomization

The location of soil incubations was fully randomized within the controlled temperature environment and measurement order was randomized.

Blinding

Soil incubations were labeled with numeric IDs and thus investigators were blind to litter treatment assignment during the incubation. However, full blinding was not possible as mineral-amended soils were visually distinct from one another. All samples were treated the same regardless of treatment allocation and data analysis was conducted on all samples simultaneously.

Did the study involve field work? ☒ Yes ☐ No

Field work, collection and transport

Field conditions	Soils were collected from a farm near Glassonby, Penrith in August 2020. The conditions were fine (~20°C) and dry
Location	54°44′27″N , 002°39′52″W
Access & import/export	Samples were collected with permission from the landowner
Disturbance	No significant disturbance was caused by the sampling of soils within the field

Reporting for specific materials, systems and methods

We require information from authors about some types of materials, experimental systems and methods used in many studies. Here, indicate whether each material, system or method listed is relevant to your study. If you are not sure if a list item applies to your research, read the appropriate section before selecting a response.

Materials & experimental systems

n/a	Involved in the study
☒	☐ Antibodies
☒	☐ Eukaryotic cell lines
☒	☐ Palaeontology and archaeology
☒	☐ Animals and other organisms
☒	☐ Clinical data
☒	☐ Dual use research of concern
☒	☐ Plants

Methods

n/a	Involved in the study
☒	☐ ChIP-seq
☒	☐ Flow cytometry
☒	☐ MRI-based neuroimaging

Plants

Seed stocks	Not applicable
Novel plant genotypes	Not applicable
Authentication	Not applicable
