# Supplementary material for: Microbial and mineral interactions decouple litter quality from soil organic matter formation
Source: Nat Commun. 2024 Nov 20;15:10063. doi: 10.1038/s41467-024-54446-0 (PMC11579368; doi:10.1038/s41467-024-54446-0)
Supplement: Supplementary file 3 — Peer Review File [file 41467_2024_54446_MOESM3_ESM.pdf]

nature portfolio

Peer Review File

Microbial and mineral interactions decouple litter quality from soil organic matter formation

Corresponding Author: Dr Jeanette Whitaker

This file contains all reviewer reports in order by version, followed by all author rebuttals in order by version.

Version 0:

Reviewer comments:

Reviewer #1

(Remarks to the Author)
This paper reports on an interesting, highly controlled laboratory study that reports on the relative effects of plant litter quality and soil mineralogy on the mechanisms of MAOM formation. As the authors point out, the widely promoted 'MEMS' hypothesis that high-quality litters promote MAOM formation is not supported by the literature, and even some studies show the opposite effect. Hence, there is great need for research such as this that explores not just the net mass transfer of litter C to MAOM-C but also the mineralogical and microbial mechanisms affecting the net transfer.

To my knowledge, the major result reported herein, the trade-off between higher CUE with low quality litter but higher priming of native MAOM compared to high-quality litter (such that there is no net effect of litter quality on MAOM), is a new finding that should generate interest. This finding is also partially explained by changes in the microbial community that occur as a result of an interaction between litter quality and mineralogy. These results will be useful to future work.
All this said, I do think there could be better presentation of and connection to previous work about litter quality effects on SOM. At present, the paper is written as if there has been little questioning of the MEMS hypothesis. However, shortly after it was published in GCB, there were several papers in GCB and SBB that summarized effects of litter quality studies on SOM formation and reported no consistent effect. So, this is well known and I think the paper exaggerates the novelty of results (this distracts from the true novelty of the mineralogy and microbiological explanations of the inconsistency). As part of a potential reframing, I think the paper would benefit from a shift in presenting the MEMS hypothesis as a 'straw man' to an explaining why the effect of litter quality on SOM formation is inconsistent (a lack of integration about litter, mineralogy, microbes, and environment, maybe more). There are hints of such an approach for example at line 160 where the % effect is attributed to different variables.

I also encourage the authors to better highlight two aspects of uncertainty about the results: 1) The potential effects of environment. As the authors suggest different mineralogy could produce different water potentials at the same volumetric or gravimetric water contents. I will add that one reason we don't observe consistent effects in the field is that plants with high quality litters tend to produce much less biomass than plants with low quality litters leading to different soil microenvironments. 2) The effect of mineralogy has been indirectly addressed through the concept of C saturation or C status effects on the efficiency of MAOM formation. These works are not mentioned but have particular relevance given the use of pristine minerals. How does the fact that the minerals have no SOM affect the results, especially in the context of C saturation theory?

Finally, can there be any inference on the relative importance of stabilization of plant- vs. microbial-derived compounds? Especially in the context of the Liang and Angst papers that are referenced. This is the main missing piece of this work. If measurements of plant vs microbial derived litter-C in MAOM were available it would be great, but should not be required as the paper already makes some significant advances. Speculation would be nice.

Minor points

Litter quality is ultimately about the kinetics of mineralization (not CN ratios or lignin, etc.). It should be described as such.
Line 83: not supporting or opposing, but different effects in different environments. How can your results help understand this inconsistency?

Line 127: when you say counteracting, do you mean that there was no net effect of litter quality on SOC? This could be clearer.

Line 141: if soil mineralogy was the strongest control on respired litter, does that mean a significant portion of the plant C compounds were directly stabilized? I think it must?
Line 148: by pre-existing, do you mean mineralization of SOC from the soil and not the added litter?

Line 168: please include the equation for the index here.

Lines 173-176 and Figure 3b: The wording is confusing to me. The figure shows a significant accumulation of MAOM-C during the incubation period. So, if there an "equivalent loss of pre-existing C from MAOM and gain of litter C stabilized as MAOM", we would expect no difference with the no-litter control?

Figure 3a: If the MAOM efficiency is ~33% higher for low vs. high quality litter, yet in Figure 3b the accumulation of MAOM almost no different for low vs. high quality…what's going on. Maybe you can explicitly say this around line 177.

Lines 222-224 most important.

Line 246: maybe, but at best an indirect factor.

Line 251: if it is true that the priming is owed to N mining, then the C:N ratio of the MAOM must decline. Was this observed?

Reviewer #2

(Remarks to the Author)
Overall Comments:
This paper evaluates the influence of litter quality, microbial community, and mineral type on SOM, and mostly MAOM, formation and loss via priming. The authors use a clever experimental design that lets them assess the effects of all three of these controls together and their influence on both MAOM formation and loss, providing a unique dataset that will surely be of interest to the soil organic matter community. Overall, they find that mineralogy is the strongest control on MAOM formation and loss and, importantly, that high quality litters both form less MAOM and do so less efficiently, contrary to current theory. They attribute this unexpected result to changes in the microbial community. Other studies have found similar MAOM responses but this work better provides mechanisms with microbial data and also contextualizes these findings with mineral effects. I believe this is a really useful paper for the SOM community that addresses outstanding questions and prompts the need for more in-situ studies of these controls to inform model formulations. Overall, I think this is a great study and is written relatively clearly for the reader. I provide largely text edits below that could increase clarity, precision, and readability.

Specific Comments:
Line 23: I would say most SOC is associated with minerals in mineral soil. The largest amount of SOC is stored in organic soils which largely consist of particulate organic matter unassociated with minerals.
Lines 24-26: There has been recent work suggesting the majority of mineral-associated SOC is plant derived (Angst et al., 2021, Chang et al., 2024) or that determining the relative contributions of plants and microbes is uncertain (Whalen et al., 2022). For that reason, I suggest the authors use less definitive language here.
Angst, G., Mueller, K.E., Nierop, K.G., Simpson, M.J., 2021. Plant-or microbial-derived? A review on the molecular composition of stabilized soil organic matter. Soil Biology and Biochemistry, 108189.
Chang, Y., Sokol, N.W., van Groenigen, K.J., Bradford, M.A., Ji, D., Crowther, T.W., Liang, C., Luo, Y., Kuzyakov, Y., Wang, J., 2024. A stoichiometric approach to estimate sources of mineral-associated soil organic matter. Global Change Biology 30, e17092.
Whalen, E.D., Grandy, A.S., Sokol, N.W., Keiluweit, M., Ernakovich, J., Smith, R.G., Frey, S.D., 2022. Clarifying the evidence for microbial-and plant-derived soil organic matter, and the path towards a more quantitative understanding. Global Change Biology.
Line 61: Again, MAOM is dominant in mineral soils – please add this.
Lines 70-71: I would rephrase this – the MEMS hypothesis specifically suggests that litter quality, through microbial anabolism, influences MAOM formation. It's really rather that previously, low litter quality was thought to confer persistence but with MEMS, high litter quality is now thought to cause more MAOM formation and confer persistence.
Lines 121-123: It would be helpful to note that both less MAOM was formed from high quality litter and it was less efficient. Some other studies have found greater MAOM formation but that it was less efficient (e.g., Cordova, 2018) so clarifying that this study did not find that would be helpful.
Cordova, C., 2018. Plant litter quality affects the accumulation rate, composition, and stability of mineral-associated soil organic matter. Soil Biology and Biochemistry.
Line 138: I would stay consistent in using MAOM formation.
Line 181-182: This data is for the soils overall correct? Rather than for the amount of isotopic label that is ingested by the microbes? If yes, it would be helpful to make that clear, since I was initially confused by the difference between this result and that at lines 163-165.
Line 206: It would be good to emphasize the switch here to talking about litter saprotrophs rather than soil saprotrophs because this distinction is easy to miss and could confuse readers (it took me two reads to recognize the difference).
Line 211: Higher relative abundance as compared to high quality litter?
Lines 238-240: I didn't see the CUE reported in the results. Could a sentence or two describing this result be added there?

Lines 240-242: This would be a good place to comment on the limitations of the method used for calculating CUE in this study. Because the authors use the 13C labeled litter to calculate CUE they can really only speak to the communities using that labeled litter and not the entire soil community in their microcosms. I don't think the statement here is incorrect but just requires clarity that the CUE measurement here can only speak to the community existing on the litters.
Lines 248-250: I don't believe the N-mining reasoning is explained in the introduction. I agree this is a logical hypothesis but should be explained more clearly in the introduction.
Lines 255-258: Why would dominance of oligotrophs cause greater priming?
Lines 270-273: I find the first half of this sentence confusing. It is unclear if the text after "with" is explaining the clause before or is another example of consistency. It may be useful to break up the sentence for clarity.
Line 290: I would suggest adding "on geothite" after "higher C retention".
Lines 291-294: I would break this up into two sentences and reorganize the second half of the sentence. I don't quite understand the second half of the sentence as it is now – are the authors highlighting that (1), (2), and (3) were more important in their study than litter quality?
Line 295: I do not think "dictating" is the right word here – maybe "influencing"?
Line 506: Please ensure it is clear in the results that these are relative importance percentages and thus do not represent the amount of total variability a given predictor explains.
All Figures: Please ensure these are color-blind friendly colors.
Figure 2: I would make the faceted plots in (A) and (B) all one color and a different color than those in (C) and (D). Currently, it seems as if the legend applies to (A) and (B) as well as (C) and (D).
Figure 3: I would not call these MAOM C stocks since the authors do not account for bulk density (nor should they in an incubation). Rather, I would call this MAOM C content.
Figures 4 and 5: Please add the legends with their respective sub-figures. Having triangles for the mineral and litter types makes it confusing when all of the legends are together.

Reviewer #3

(Remarks to the Author)
The manuscript by Elias et al. presents results from a laboratory incubation in which a soil was amended with different minerals and 13C labeled litter of different quality. The aim of the study was to test the prevailing concept that high-quality, in contrast to low-quality, litter fosters MAOM formation. The authors fractionated the soil into POM and MAOM, monitored heterotrophic respiration, and traced the 13C label in fractions, respired CO2, and microbial biomass. They also analyzed the microbial community via DNA extractions. The authors found more litter-derived C in MAOM in the low-quality than high-quality litter treatment, with little effects on overall C in MAOM. They explain this by litter-quality induced shifts to a microbial community with lower CUE and priming of preexisting SOM. Notably, mineralogy determined most of the variation in litter-derived C in MAOM. The study is timely, well-written, and the methodology appears to be sound. I have two major, and many moderate and minor, comments that need to be addressed.
1. One of the main conclusions is that mineralogy more strongly governed MAOM formation than litter chemistry. This may have simply been due to the addition of clean mineral surfaces without any SOM attached. This is hardly (if at all) encountered under natural conditions. So mineralogy may have a substantially weaker effect in soils in which part or all of the mineral surfaces present are covered with SOM. This limitation hampers the transferability of the study's results to natural systems and needs to be discussed to put the results into context.
2. POM was isolated by the authors, but effects of litter chemistry or mineralogy on this fraction are not discussed, although it can be important to C sequestration as well.

More comments below:
The abstract would benefit from the placement of commas.
L25 Whether MAOM is dominated by microbial compounds is highly context-specific, so I suggest rephrasing this sentence.
L61ff The authors could refer to studies that show that MAOM can also cycle on much shorter timescales than indicated in these lines.
L70ff This statement contradicts those in the abstract and below, i.e., that high-quality litters are considered to boost MAOM formation (sensu Cotrufo et al., 2013).
L86ff What about effects of litter quality on POM, which can be a substantial store of C as well?
L97ff Occlusion and aggregation rather influence the stability of POM, if not considering particles <50 µm. The sentence is further written confusingly as it indicates that adsorption, occlusion, and aggregation form MAOM, which is probably not the meaning the authors want to convey. I suggest rephrasing the sentence for clarity.
L114ff Did the authors hypothesize that mineralogy is more important than litter quality? This does not become clear here.
L119ff This comes surprising. I suggest incorporating information on how microbial community composition can affect SOC stability and, perhaps, also include a hypothesis on this.
L121ff The magnitude of the difference in MAOM formation between treatments amended with high- and low-quality litters could become clearer in this passage, as both litters appeared to have detrimental or no effects on overall MAOM formation.
L123ff This sentence is very unspecific. How did litter quality shift microbial communities?
L125ff Did the authors measure the related data or is this statement purely speculative?

L135ff Did the authors measure the SSA of the final soil-mineral mixture? This would somehow be more informative than the SSA of the individual minerals since the SSA of the soil-mineral mixture eventually determines reactivity.
L281ff This statement is purely speculative and not supported by the results.

L308ff How was litter quality determined?
L318ff Given the authors sampled soil from an agricultural field, were there carbonates? I was wondering because the authors refer to SOC throughout, which might be incorrect when inorganic C was present in the soil.
L340ff How were minerals mixed with the soils?
L371/372 Why this incubation time?
L417 50 W is the energy output of the instrument, but this does not provide any information on which energy reached the soil. I suggest calculating the energy that was delivered to the soil-SPT mixture in J/ml, as common in related studies.
L426ff Given the method well separated fractions, the fraction remaining on the 53-µm sieve should be sand only and no POM.

Version 1:

Reviewer comments:

Reviewer #1

(Remarks to the Author)
The authors did a nice job of addressing my review and I don't have any further urgent comments. I think the paper deserves to be published in Nature Comm. It makes a significant advance in our understanding about the uncertain effect of litter quality on SOM stabilization – this is a question people have been pursuing for decades.

Reviewer #3

(Remarks to the Author)
The authors mosly well responded to my comments. However, I still have concerns regarding the fractionation (see below).

Newly added text in L71-74: I am not sure whether mentioning recalcitrance in this context is helpful.
Newly added text in L74-77: I suggest adding "can" before "form" since microbial necromass in, e.g., forests can be as low as ~10%.
Newly added text in L119-121: Is not rather the molecular interaction of organic matter and minerals desicive for the availability of that organic matter rather than reduced concentrations of DOC? I suggest rephrasing the sentence.
L102-105 Authors may also refer to recent studies on how microbial diversity can affect necromass formation partly independently of CUE.
Reply to my comment on L135ff: The info given in the reply could be added to the manuscript, for clarity.
L417 sonication energy: The delivered energy appears to be very low (see e.g., Just et al. https://doi.org/10.3389/fsoil.2021.692583). It is thus questionable whether the authors properly dispersed the soil. Why was this low energy output chosen? This does not hamper comparability among samples in the current study, but it is questionable whether the results can be compared with those of other studies or transferred to properly dispersed soils. I acknowledge that sandy loam may require lower energies than clay-rich soils; however, even sandy loam can contain ~20% of clay, for which the chosen energy appears to be low.
Reply to my comment on L426ff: The authors separated fPOM and oPOM via density using SPT. As such, the fraction on top of the 53-µm sieve after separtion of POM should be sand only without any POM at all, given that the authors' separation was effective. It does not make sense to then again combine the presumably clean POM fractions the authors isolated via density with the mineral sand fraction on the 53-µm sieve (as indicated in L527), as use of SPT would not have been needed in the first place. The description in the methods is either confusing or the authors have not understood the rationale for separating POM via SPT.

Reviewer #4

(Remarks to the Author)
Overall comments: I appreciate the authors attention to my comments and those of the other reviewers. I am mostly satisfied with the resubmitted manuscript and am excited for it to contribute to the literature. The only bigger concern I have with the resubmitted manuscript is the very low CUE reported in this experiment (largely below 0.1). While this CUE isn't outside of the realm of possibility, these low values certainly deserve some speculation about why they are so low and some defense from the authors.

Other minor comments:
Line 67: POM turns over faster on average – see Heckman, Hicks-Pries et al., 2022 for overlapping turnover times of POM and MAOM.
Heckman, K., Hicks Pries, C.E., Lawrence, C.R., Rasmussen, C., Crow, S.E., Hoyt, A.M., von Fromm, S.F., Shi, Z., Stoner, S., McGrath, C., Beem-Miller, J., Berhe, A.A., Blankinship, J.C., Keiluweit, M., Marin-Spiotta, E., Monroe, J.G., Plante, A., Schimel, J., Sierra, C.A., Thompson, A., Wagai, R., 2022. Beyond bulk: Density fractions explain heterogeneity in global soil carbon abundance and persistence. Global change biology 28, 1178-1196.
Lines 78-94: The empirical evidence for MEMS and for the Castellano et al., 2015 paper are reviewed in Rocci et al., 2024 if the authors want a more recent review of the evidence for these therories.
Rocci, K., Cotrufo, M.F., Ernakovitch, J., Foster, E., Frey, S., Georgiou, K., Malhotra, A., Reich, P., Schlerman, E., Wieder, W.R., 2024. Bridging 20 years of soil organic matter frameworks: empirical support, model representation, and next steps. Journal of Geophysical Research Biogeosciences.
Line 93: I feel the mention of SOC models here is too brief to be usedul and I suggest remocing it. Particularly, it is unclear

what “current generation” models are? Are these microbially implicit models?
Lines 109-111: I would also cite more fundamental nutrient mining papers here such as Moorhead and Sinsabaugh, 2006 and Chen et al., 2014, where Chen et al., 2014 also found that oligotrophs drive the priming effect.
Chen, R., 2014. Soil C and N availability determine the priming effect: microbial N mining and stoichiometric decomposition theories. Global Change Biology 20, 2356–2367, doi: 10.1111/gcb.12475
Moorhead, D.L., Sinsabaugh, R.L., 2006. A theoretical model of litter decay and microbial interaction. Ecological Monographs 76, 151-174.
Lines 255-257: Is this line different from the preceding one because the specialist saprotrophs are not influenced by litter quality?

Version 2:

Reviewer comments:

Reviewer #3

(Remarks to the Author)
The authors have tackled my comments well. However, I am still not convinced of "sand-sized SOM."

I am not aware of any study that has shown that the "sand-sized fraction can represent organic debris encrusted in fine minerals," and without appropriate citations or experimental evidence, this statement is not credible, as the C in the sand fraction can simply be an artifact of the fractionation procedure.

I thus suggest either providing references/experimental evidence for the statement above or not discussing sand-sized SOM in the manuscript.

Open Access This Peer Review File is licensed under a Creative Commons Attribution 4.0 International License, which permits use, sharing, adaptation, distribution and reproduction in any medium or format, as long as you give appropriate credit to the original author(s) and the source, provide a link to the Creative Commons license, and indicate if changes were made.
In cases where reviewers are anonymous, credit should be given to 'Anonymous Referee' and the source.
The images or other third party material in this Peer Review File are included in the article’s Creative Commons license, unless indicated otherwise in a credit line to the material. If material is not included in the article’s Creative Commons license and your intended use is not permitted by statutory regulation or exceeds the permitted use, you will need to obtain permission directly from the copyright holder.
To view a copy of this license, visit https://creativecommons.org/licenses/by/4.0/

Response to Reviewers

REVIEWER COMMENTS

Reviewer #1 (Remarks to the Author):

This paper reports on an interesting, highly controlled laboratory study that reports on the relative effects of plant litter quality and soil mineralogy on the mechanisms of MAOM formation. As the authors point out, the widely promoted 'MEMS' hypothesis that high-quality litters promote MAOM formation is not supported by the literature, and even some studies show the opposite effect. Hence, there is great need for research such as this that explores not just the net mass transfer of litter C to MAOM-C but also the mineralogical and microbial mechanisms affecting the net transfer.

To my knowledge, the major result reported herein, the trade-off between higher CUE with low quality litter but higher priming of native MAOM compared to high-quality litter (such that there is no net effect of litter quality on MAOM), is a new finding that should generate interest. This finding is also partially explained by changes in the microbial community that occur as a result of an interaction between litter quality and mineralogy. These results will be useful to future work.

We thank Reviewer #1 for their positive comments regarding the manuscript.

All this said, I do think there could be better presentation of and connection to previous work about litter quality effects on SOM. At present, the paper is written as if there has been little questioning of the MEMS hypothesis. However, shortly after it was published in GCB, there were several papers in GCB and SBB that summarized effects of litter quality studies on SOM formation and reported no consistent effect. So, this is well known and I think the paper exaggerates the novelty of results (this distracts from the true novelty of the mineralogy and microbiological explanations of the inconsistency). As part of a potential reframing, I think the paper would benefit from a shift in presenting the MEMS hypothesis as a 'straw man' to an explaining why the effect of litter quality on SOM formation is inconsistent (a lack of integration about litter, mineralogy, microbes, and environment, maybe more). There are hints of such an approach for example at line 160 where the % effect is attributed to different variables.

We thank Reviewer #1 for highlighting the lack of discussion around the inconsistent evidence to support the MEMS hypothesis. To address this shortcoming, the introduction has been reframed to focus more on the mechanisms that may explain the inconsistent effects of litter quality on SOM formation observed across studies as detailed below.

L83-105 *"Microbial CUE tends to decline as soil nutrient availability decreases and thus, the MEMS framework proposes that MAOM should be formed more efficiently (more MAOM formed, and less CO2 produced per unit of litter C) in high quality litter environments compared to low quality litter environments. However, a review of controlled experiments suggests that the effect of litter quality on SOM stabilisation is inconsistent, and that high quality litter is not always stabilised more efficiently by the mineral soil matrix relative to low quality litter. Moreover, the litter quality effect varies across studies conducted under different environmental conditions and different soil types. To resolve uncertainties between current generation and new microbial explicit SOC models requires greater consideration of the interactions between litter quality, microbes, the mineral matrix, and the abiotic environment.*

Litter-microbial interactions are one mechanism proposed to explain why the effect of litter quality on SOM is inconsistent. For example, although low-quality litter may be mineralized with low microbial CUE by individual taxa, adaptation at the community level to overcome nutrient limitation may allow for decomposition without a reduction in community-level CUE."

Changes to the introduction have also been made highlighting how mineralogy may explain the inconsistent relationship between litter quality and SOM with reference to the soil C saturation concept.

L116-130 *"The effect of litter quality on SOM may also vary under differing abiotic conditions, such as soils of varied mineralogy. While soil microbial processes produce organic compounds through microbial anabolism, the soil mineral matrix acts as the long-term control on SOM content and stability in soils. However, not all minerals are equivalent in their ability to stabilise OC. Variation in mineral specific surface area (SSA) and surface charge influences adsorption of organic molecules, which limits microbial access by reducing dissolved organic C concentrations in soils, while clay minerals (e.g. phyllosilicates such as low-activity kaolinite and high-activity montmorillonite) and metal (oxyhydr)oxides can also catalyse polymerization of organic molecules conferring stability. Mineralogy may also control the inherent maximum capacity of soils to store mineral-associated SOC (the C saturation concept) as variation in specific surface area (SSA) controls the finite amount of reactive mineral surfaces available for C retention. The C saturation deficit of a given soil (proximity to their maximum carbon-storage capacity) may influence the rate of C accumulation, thus modulating the relationship between litter quality and SOM."*

The exploration of why the effects of litter quality on SOM are inconsistent have also been made central to the objective of the study.

L134-136 "*Here we investigated the interactive effects of litter quality, soil mineralogy and soil microbiology on the formation of MAOM, under highly controlled conditions to explore the mechanisms behind inconsistent effects of litter quality on SOM."*

The concluding paragraph of the discussion has been rephrased to refer back to the inconsistent effects of litter quality on SOM.

L389-391 "*However, our results suggest that inconsistency in the effect of litter quality on MAOM accumulation is driven by differences in: the soil mineral matrix; microbial responses to litter additions; and priming of pre-existing SOM……."*

I also encourage the authors to better highlight two aspects of uncertainty about the results: 1) The potential effects of environment. As the authors suggest different mineralogy could produce different water potentials at the same volumetric or gravimetric water contents. I will add that one reason we don't observe consistent effects in the field is that plants with high quality litters tend to produce much less biomass than plants with low quality litters leading to different soil microenvironments. 2) The effect of mineralogy has been indirectly addressed through the concept of C saturation or C status effects on the efficiency of MAOM formation. These works are not mentioned but have particular relevance given the use of pristine minerals. How does the fact that the minerals have no SOM affect the results, especially in the context of C saturation theory?

Thank you for highlighting these uncertainties.

1) We agree that environmental interactions are a significant source of uncertainty as mineralogy can also strongly influence soil abiotic conditions. Therefore, whether the effect of mineralogy on SOM dynamics is due to variation in mineral chemistry/reactivity or mineral-environmental-microbial interactions is difficult to disentangle. Text has been added to the discussion expanding upon this point and highlighting variation in production rates across litters under field conditions.

L374-378 "*However, a key uncertainty remains concerning the relative importance of mineral chemistry itself, against indirect mineral effects on the soil abiotic environment in shaping microbial community responses. Nonetheless, our findings suggest that the effect of mineralogy on SOM dynamics may not be solely abiotic, and that microbe-mineral interactions could modify the effect of litter quality on MAOM formation efficiency."*

L386-391 *"Our highly controlled laboratory incubation of amended soils does not include the full range of processes that occur under real-world conditions. For example, litter quality is also confounded with variation in production rates, which may lead to different soil microenvironments. However, our results suggest that inconsistency in the effect of litter quality on MAOM accumulation is driven by differences in: the soil mineral matrix; microbial responses to litter additions; and priming of pre-existing SOM"*

2) Thank you for highlighting this omission. MAOM exhibits saturation dynamics due to the finite availability of mineral surfaces (Cotrufo et al, 2019). Therefore, soils with a greater C saturation deficit may be expected to have higher MAOM formation efficiency (Mitchell et al, 2021, Stewart et al, 2008, Stewart et al, 2007). The addition of pristine minerals increased the mineral surface area of the soils by approximately 26 – 93% (based on simple mixing ratios at 10% w/w additions) (Control soil = 3.10 m^2/g, G-amended soil = 3.91 m2/g , K-amended soil = 3.80 m/g, M-amended soil = 5.97 ± 0.01 m2/g), effectively increasing their C saturation deficits as all mineral surfaces were initially available for organo-mineral interactions. Montmorillonite has approximately three times more surface area relative to kaolinite and goethite and thus, addition by mass at 10% w/w likely resulted in differing initial C saturation deficits between mineral treatments (although see actual measured SSA of soil-mineral mixtures below in comments to Reviewer 3). In agreement with the prediction that MAOM formation efficiency is higher at greater C saturation deficits we found that montmorillonite amended soils formed the most litter derived MAOM. However, we also observed the greatest litter quality effect in montmorillonite amended soils (presumably with the largest initial C saturation deficit). It has been proposed that in soils with especially large C saturation deficits, the conversion of litter-C to MAOM is high regardless of litter chemistry – and that the effect of litter quality on mineral-associated SOM may be most easily observed at moderate C saturation deficits (Castellano et al, 2015). Text has been added to the discussion to highlight the uncertainty regarding use of pristine minerals and the potential effects of variable soil C saturation deficits on MAOM formation efficiency between soils.

Mitchell, E. et al. Important constraints on soil organic carbon formation efficiency in subtropical and tropical grasslands. Global Change Biology 27, 5383-5391 (2021). https://doi.org/https://doi.org/10.1111/gcb.15807

Cotrufo, M. F., Ranalli, M. G., Haddix, M. L., Six, J. & Lugato, E. Soil carbon storage informed by particulate and mineral-associated organic matter. Nature Geoscience 12, 989-994 (2019). https://doi.org/10.1038/s41561-019-0484-6

Castellano, M. J., Mueller, K. E., Olk, D. C., Sawyer, J. E. & Six, J. Integrating plant litter quality, soil organic matter stabilization, and the carbon saturation concept. Global Change Biology 21, 3200-3209 (2015). https://doi.org/https://doi.org/10.1111/gcb.12982

Stewart, C. E., Paustian, K., Conant, R. T., Plante, A. F. & Six, J. Soil carbon saturation: Evaluation and corroboration by long-term incubations. Soil Biology and Biochemistry 40, 1741-1750 (2008). https://doi.org/https://doi.org/10.1016/j.soilbio.2008.02.014

Stewart, C. E., Paustian, K., Conant, R. T., Plante, A. F. & Six, J. Soil carbon saturation: concept, evidence and evaluation. Biogeochemistry 86, 19-31 (2007). https://doi.org/10.1007/s10533-007-9140-0

L347-361 *"We acknowledge that our results may be influenced by applying pristine minerals to soils, as most mineral surfaces in natural systems are already associated with organic molecules. However, a study by Pronk et al. (2012) using artificial soils found substantial sorption of OM to fresh mineral surfaces within a few days. Therefore, pristine minerals added to soils may rapidly form organo-mineral associations, that more closely resemble those in natural soils. Nonetheless, caution should be exercised when translating these findings to natural systems. The finding that montmorillonite soils formed new MAOM from plant litter more efficiently than kaolinite soils may also be due to a greater C saturation deficit (due to different SSA of pristine minerals added to soils) as MAOM exhibits saturation dynamics due to the finite availability of mineral surfaces (Cotrufo et al, 2019). Our findings agree with previous work showing that MAOM formation efficiency is strongly governed by the soil C saturation status with the highest efficiency at the greatest C saturation deficit (Mitchell et al, 2021, Stewart et al, 2008, Stewart et al, 2007). It has also been proposed that in soils with high C saturation deficits the conversion rate of litter-C to mineral-associated SOC is high, regardless of litter quality[30]. However, we observed the greatest litter quality effect in montmorillonite amended soils with the greatest C saturation deficit."*

Finally, can there be any inference on the relative importance of stabilization of plant- vs. microbial-derived compounds? Especially in the context of the Liang and Angst papers that are referenced. This is the main missing piece of this work. If measurements of plant vs microbial derived litter-C in MAOM were available it would be great, but should not be required as the paper already makes some significant advances. Speculation would be nice.

We agree that partitioning of ^{13}C stabilised as MAOM into plant and microbially derived compounds would allow for an assessment of the relative importance of direct sorption and indirect, microbially mediated stabilisation mechanisms. Unfortunately, we did not measure biomarkers that could distinguish between plant and microbially derived MAOM and there are known limitations with current available methods. We speculate that as mineralogy was the strongest control on respired litter C, this could indicate that direct sorption of litter C was an important mechanism in our microcosms, limiting microbial respiration of litter-C by reducing concentrations of soluble litter compounds in the soil (Also see response to minor point L141 below). Speculation on the balance of direct vs indirect stabilisation has been added to the discussion and the value of quantifying these two pathways using biomarkers in future work has been discussed.

L324-336 *"In agreement with our second hypothesis, that soil mineralogy would control the formation of litter-derived MAOM more than litter quality (H2), we found that soil mineralogy explained the majority of variation in litter-C respired (57.2 %) and litter-C stabilised in MAOM (63 %), whilst the effect of litter type was smaller (20.8 %) and dependent on soil mineralogy (Figure 1A,*

Figure 2A, Table S1-S2). This suggests that a significant proportion of plant C compounds may have been directly stabilised through sorption. However, it could also reflect microbe-mineral interactions that shifted microbial community composition and physiology, promoting biomass production (efficiency) and stabilization of resultant microbial products by association with soil minerals. *We did not measure biomarkers in MAOM to quantify plant and microbial derived SOM and there are known limitations with these methods. However, a recent meta-analysis suggests that whilst microbial pathways of SOM formation are important, ~50 % of MAOM-C may be plant-derived. Therefore, improving quantification of plant and microbially derived SOM is a key research need."*

Minor points

Litter quality is ultimately about the kinetics of mineralization (not CN ratios or lignin, etc.). It should be described as such..

L71-75 Replaced high or low C:N, and high or low lignocellulose index with low or high mineralization rate.

Line 83: not supporting or opposing, but different effects in different environments. How can your results help understand this inconsistency?

L91-94 Changed to "…*the litter quality effect varies across studies conducted under different environmental conditions and different soil types. To resolve uncertainties between current generation and new microbial explicit SOC models requires greater consideration of the interactions between litter quality, microbes, the mineral matrix, and the abiotic environment."*

Line 127: when you say counteracting, do you mean that there was no net effect of litter quality on SOC? This could be clearer.

L163-166 "Rephrased for clarity. *"Addition of low-quality litter enhanced mineralisation of pre-existing SOC decomposition (not derived from added litter), counteracting the positive effect of low-quality litter on the efficiency of MAOM formation."*

Line 141: if soil mineralogy was the strongest control on respired litter, does that mean a significant portion of the plant C compounds were directly stabilized? I think it must?

The fact that soil mineralogy was the strongest control on respired litter C and MAOM formation may indeed suggest that a significant proportion of plant C compounds were directly stabilised. However, it may also reflect microbe-mineral interactions that shifted microbial community composition and their physiology, promoting biomass production (efficiency) and greater retention of microbial products. Although litter quality was the strongest driver of shifts in microbial communities, minerals themselves also played a role. Text has been added to the discussion.

"…This suggests that a significant proportion of plant C compounds may have been directly stabilised through sorption. However, it could also reflect microbe-mineral interactions that shifted microbial community composition and physiology, promoting biomass production (efficiency) and stabilization of resultant microbial products by association with soil minerals" **L328-332**

Line 148: by pre-existing, do you mean mineralization of SOC from the soil and not the added litter?

Yes. This is now clarified at **L137-138** where pre-existing is first mentioned. *"…changes in MAOM formation and priming of pre-existing SOM (present before litter addition) were influenced…"*

Line 168: please include the equation for the index here.

Equation added in text at **L207-211**. "*To account for the different decomposition rates of low- and high-quality litter (Figure 1A) an index of MAOM formation efficiency was calculated (MAOM Formation Efficiency = MAOM-C_{litter} / (MAOM-C_{litter} + Respired C_{litter}) where MAOM-C_{litter} is the total litter-C recovered in the MAOM fraction and Respired C_{litter} is the litter-C respired throughout the experiment)*". This is also described in more detail in the methods at **L592-598.**

Lines 173-176 and Figure 3b: The wording is confusing to me. The figure shows a significant accumulation of MAOM-C during the incubation period. So, if there an "equivalent loss of pre-existing C from MAOM and gain of litter C stabilized as MAOM", we would expect no difference with the no-litter control?

Thank you for highlighting this ambiguity. Here, reference is being made to the comparison between low- and high-quality litter. Litter addition, irrespective of quality increased MAOM-C content relative to no litter controls (Figure 3B). However, despite low-quality litter forming new MAOM more efficiently (See Figures 2A & 3A), total MAOM-C content (including both litter derived C and pre-existing SOC present before litter addition) was not different between low- and high-quality litter treatments (Figure 3B). We hypothesise that this is due to the greater priming of SOC present before litter addition from low-quality litter treatments shown in Figure 1B. This has been re-worded for clarity as below.

"Litter addition increased the total MAOM-C content in microcosms (including C derived from litter additions and pre-existing SOC present before litter addition) relative to no litter controls (Figure 3B). However, microcosm total MAOM-C content did not differ between low- and high-quality litter in no mineral, kaolinite, and goethite soils (Figure 3B, Table S3). This indicates that more pre-existing MAOM-C was lost in low- relative to high-quality litter treatments, counterbalancing the effect of litter quality on litter-C transfer to MAOM (Figure 3B, Table S3). This is supported by increased priming of pre-existing SOC in low- relative to high-quality litter treatments (Figure 1B). However, total MAOM-C content was marginally greater (p=0.08) in low- relative to high-quality litter in montmorillonite soil (Figure 3B, Table S3)." **L216-226**

Figure 3a: If the MAOM efficiency is ~33% higher for low vs. high quality litter, yet in Figure 3b the accumulation of MAOM almost no different for low vs. high quality…what's going on. Maybe you can explicitly say this around line 177.

Low-quality litter formed new MAOM more efficiently (See Figures 2A & 3A), however total MAOM-C content (including both litter derived C and pre-existing SOC present before litter addition) was not different between low- and high-quality litter treatments (Figure 3B). We hypothesise that this is due to the greater priming of SOC present before litter addition from low-quality litter treatments as shown in Figure 1B. Please see comment above that addresses this.

Lines 222-224 most important.

We agree that it is an important point that other studies do support our findings. We have highlighted these and the novelty of our work that incorporates microbial responses to litter quality.

Line 246: maybe, but at best an indirect factor.

Text amended to *"that microbial CUE is a major, although indirect, determinant of global soil C storage"* **L304-305**

Line 251: if it is true that the priming is owed to N mining, then the C:N ratio of the MAOM must decline. Was this observed?

The CN ratio of MAOM was higher in low- relative to high-quality litter treatments. This does suggest that greater priming following low quality litter addition may be explained by enhanced N mining from pre-existing MAOM. The C, N and C:N ratio of MAOM has been added to Supplementary Information: Table S7 and the following sentence has been added to the discussion.

"The C:N ratio of MAOM was higher in low- relative to high-quality litter which does support the N mining hypothesis (Table S7)". **L314-315**

Reviewer #2 (Remarks to the Author):

Overall Comments:
This paper evaluates the influence of litter quality, microbial community, and mineral type on SOM, and mostly MAOM, formation and loss via priming. The authors use a clever experimental design that lets them assess the effects of all three of these controls together and their influence on both MAOM formation and loss, providing a unique dataset that will surely be of interest to the soil organic matter community. Overall, they find that mineralogy is the strongest control on MAOM formation and loss and, importantly, that high quality litters both form less MAOM and do so less efficiently, contrary to current theory. They attribute this unexpected result to changes in the microbial community. Other studies have found similar MAOM responses but this work better provides mechanisms with microbial data and also contextualizes these findings with mineral effects. I believe this is a really useful paper for the SOM community that addresses outstanding questions and prompts the need for more in-situ studies of these controls to inform model formulations. Overall, I think this is a great study and is written relatively clearly for the reader. I provide largely text edits below that could increase clarity, precision, and readability.

We thank Reviewer #2 for their positive and constructive feedback.

Specific Comments:

Line 23: I would say most SOC is associated with minerals in mineral soil. The largest amount of SOC is stored in organic soils which largely consist of particulate organic matter unassociated with minerals.

This opening sentence has been removed as the abstract was amended to fit within 150 words.

Lines 24-26: There has been recent work suggesting the majority of mineral-associated SOC is plant derived (Angst et al., 2021, Chang et al., 2024) or that determining the relative

contributions of plants and microbes is uncertain (Whalen et al., 2022). For that reason, I suggest the authors use less definitive language here.

Angst, G., Mueller, K.E., Nierop, K.G., Simpson, M.J., 2021. Plant-or microbial-derived? A review on the molecular composition of stabilized soil organic matter. Soil Biology and Biochemistry, 108189.

Chang, Y., Sokol, N.W., van Groenigen, K.J., Bradford, M.A., Ji, D., Crowther, T.W., Liang, C., Luo, Y., Kuzyakov, Y., Wang, J., 2024. A stoichiometric approach to estimate sources of mineral-associated soil organic matter. Global Change Biology 30, e17092.

Whalen, E.D., Grandy, A.S., Sokol, N.W., Keiluweit, M., Ernakovich, J., Smith, R.G., Frey, S.D., 2022. Clarifying the evidence for microbial-and plant-derived soil organic matter, and the path towards a more quantitative understanding. Global Change Biology.

We agree that there is still substantial uncertainty with regards to the relative contribution of microbial and plant-derived compounds to MAOM. The abstract has been amended to fit within the word limit but the remaining reference to microbial residue formation has been amended accordingly to be less definitive. *"Due to more efficient microbial anabolism, high-quality litter may produce more microbial residues for stabilisation on minerals."* **L28-30**

Line 61: Again, MAOM is dominant in mineral soils – please add this.

Text amended to "in mineral soils globally" **L61**

Lines 70-71: I would rephrase this – the MEMS hypothesis specifically suggests that litter quality, through microbial anabolism, influences MAOM formation. It's really rather that previously, low litter quality was thought to confer persistence but with MEMS, high litter quality is now thought to cause more MAOM formation and confer persistence.

Text amended to *"Litter chemistry, in particular the C:N ratio is a primary control on litter decomposition rates[19], with low-quality litter (defined as litter with low mineralization rates) promoting POM formation[16,17]. Litter quality was previously thought to be an important control on SOM persistence by selective preservation of recalcitrant organic compounds[20-22].However, high quality litter (defined as litter with high mineralization rates) is now thought to promote MAOM formation and persistence, by maximising the synthesis of microbial products that form a substantial proportion of MAOM."* **L71-77**

Lines 121-123: It would be helpful to note that both less MAOM was formed from high quality litter and it was less efficient. Some other studies have found greater MAOM formation but that it was less efficient (e.g., Cordova, 2018) so clarifying that this study did not find that would be helpful.
Cordova, C., 2018. Plant litter quality affects the accumulation rate, composition, and stability of mineral-associated soil organic matter. Soil Biology and Biochemistry.

Text amended to *"However high-quality litter formed less new MAOM, and its formation was less (not more) efficient"* **L156-157**

A reference to the Cordova et al, 2018 paper has also been added to the first paragraph of the discussion.

“Córdova, et al. (2018) also found that whilst high-quality litter formed more MAOM, its formation was less efficient relative to low quality litter.” **L273-274**

Line 138: I would stay consistent in using MAOM formation.

Text amended to MAOM formation at **L34** in abstract and **L165-166**.

Line 181-182: This data is for the soils overall correct? Rather than for the amount of isotopic label that is ingested by the microbes? If yes, it would be helpful to make that clear, since I was initially confused by the difference between this result and that at lines 163-165.

Text amended to clarify that here we are referring to the total concentration of microbial biomass, not just that derived from the added litter.

“Litter additions significantly increased the total amount of microbial biomass in soils at early (T15) and late-stage (T126) decomposition (Table 2, Table S4). Averaged across soils, total microbial biomass carbon was greater in high- relative to low-quality litter treatments during early-stage decomposition (p = 0.006).” **L228-231**

Line 206: It would be good to emphasize the switch here to talking about litter saprotrophs rather than soil saprotrophs because this distinction is easy to miss and could confuse readers (it took me two reads to recognize the difference).

The terminology of generalist soil saprotrophs and specialist litter saprotrophs was included in the figure legends for Fig. 5 and Fig. S2 so we have added those terms in on first mention in the sentences on **L253, 255, 258** to highlight this distinction.

Line 211: Higher relative abundance as compared to high quality litter?

Text amended to make clear that it is higher abundance in high compared to low-quality litter treatments.

“At late-stage decomposition, the relative abundance of generalist soil saprotrophs was still greater in soils with high- compared to low-quality litter, across all mineral treatments (Figure 5B) and were dominated by fast-growing generalist taxa from the genera Mortierella (Figure S4).” ***L257-260***

Lines 238-240: I didn’t see the CUE reported in the results. Could a sentence or two describing this result be added there?

The following sentence was added. *“Microbial CUE in soils with high-quality litter was approximately half that of the low-quality litter treatments (average of 0.03 and 0.07 respectively; Table 2).”* **L294-295**

Lines 240-242: This would be a good place to comment on the limitations of the method used for calculating CUE in this study. Because the authors use the 13C labeled litter to calculate CUE they can really only speak to the communities using that labeled litter and not the entire soil community in their microcosms. I don’t think the statement here is incorrect but just requires clarity that the CUE measurement here can only speak to the community existing on the litters.

We have added the following caveats to the text.

"*We caution that the use of ^{13}C litter tracing to calculate CUE is influenced by microbial exudation and turnover, especially over longer incubations and will likely reflect a subset of the microbial community active on the litter substrate". ***L298-300**

Lines 248-250: I don't believe the N-mining reasoning is explained in the introduction. I agree this is a logical hypothesis but should be explained more clearly in the introduction.

Text has been added to the introduction explaining why low-quality litter may promote N-mining and influence the magnitude of positive priming.

"….a recent global synthesis found that agricultural soils receiving more low-quality litter exhibited higher positive priming. Decreased soil N availability was strongly associated with the magnitude of priming suggesting accelerated nutrient mining of litter decomposers from SOM in response to low-quality litter inputs". **L107-111**

Lines 255-258: Why would dominance of oligotrophs cause greater priming?

The intensity of priming may depend on the type of microbial populations that are stimulated by the added fresh organic matter (Thomson et al, 2013, Fontaine et al, 2003). r-strategists (copiotrophs) grow rapidly in response to simple substrate availability but generally lack the ability to mineralise complex SOM. In contrast, K-strategists (oligotrophs) have wider enzymatic capabilities and can feed on SOM. The addition of complex low-quality litter with greater abundance of polymerised substances may, over time increase populations of K-strategists and the amount of SOM decomposing enzymes in the soil leading to a priming effect. We did not measure soil enzymes in this study but are speculating that the differences in microbial community composition, bacterial copy number and microbial CUE between litter treatments indicate changes to oligotroph/copiotroph (K-/r-strategists) ratios, which might also explain the divergent priming effects between litter treatments. This rationale has been clarified in the text.

*"It may also reflect observed litter-associated microbial community responses as the intensity of priming may depend on the type of microbial populations stimulated by litter addition[1]. Others have shown that the addition of complex leaf litter causes a higher bacterial oligotroph/copiotroph (K-/r-strategists) ratio, enhancing SOM decomposing enzyme activity and leading to a far greater priming effect than when simple substrates were added [69-70]" **L315-320***

Thomson, B. C. et al. Plant soil interactions alter carbon cycling in an upland grassland soil. Frontiers in Microbiology 4 (2013). https://doi.org/10.3389/fmicb.2013.00253

Fontaine, S., Mariotti, A. & Abbadie, L. The priming effect of organic matter: a question of microbial competition? Soil Biology and Biochemistry 35, 837-843 (2003). https://doi.org/https://doi.org/10.1016/S0038-0717(03)00123-8

Lines 270-273: I find the first half of this sentence confusing. It is unclear if the text after "with" is explaining the clause before or is another example of consistency. It may be useful to break up the sentence for clarity.

We agree that this sentence was unclear and this section has been revised for clarity.

This agrees with other studies of organo-mineral formation where artificial soils were created with pristine minerals and is also consistent with the greater adsorption capacity of 2:1 vs 1:1

phyllosilicates. 2:1 phyllosilicates have higher specific surface area (SSA) and more frequent isomorphic cation substitutions relative to 1:1 phyllosilicates, leading to negative surface charge. This promotes the adsorption of organic molecules through cation bridging. ***L342-346***

Line 290: I would suggest adding "on goethite" after "higher C retention".

Text amended as suggested. **L385**

Lines 291-294: I would break this up into two sentences and reorganize the second half of the sentence. I don't quite understand the second half of the sentence as it is now – are the authors highlighting that (1), (2), and (3) were more important in their study than litter quality?

This sentence has been revised for clarity and to reframe the study to focus on the inconsistent effects of litter quality on SOM formation as proposed by Reviewer #1:

"Our highly controlled laboratory incubation of amended soils does not include the full range of processes that occur under real-world conditions. For example, litter quality is also confounded with variation in production rates, which may lead to different soil microenvironments. However, our results suggest that inconsistency in the effect of litter quality on MAOM accumulation is driven by differences in: the soil mineral matrix; microbial responses to litter additions; and priming of pre-existing SOM" ***L386-391***

Line 295: I do not think "dictating" is the right word here – maybe "influencing"?

Text amended as suggested. **L392**

Line 506: Please ensure it is clear in the results that these are relative importance percentages and thus do not represent the amount of total variability a given predictor explains.

The relative importance percentages presented are not normalized to sum to 100% and rather sum to the total model R^2. Thus, the percentages presented throughout do represent the total amount of variability a given predictor alone is able to explain. This has been clarified in the statistical methods.

"To quantify the relative importance of soil mineralogy and litter quality as predictors of MAOM-C content and address H2, a metric of relative importance (lmg) was calculated, partitioning R^2 and averaging over the order of regressors using the relaimpo R package. These metrics are presented as percentages of total variance, which sum to the total model R^2." ***L607-611***

Where relative importance percentages are referred to in-text these have been labelled as "*total variability explained"* by the variable in question. Supplementary table headings have also been edited for clarity and legends have been expanded to describe the calculation of the relative importance metric presented.

All Figures: Please ensure these are color-blind friendly colors.

Thank you for highlighting this issue with the figure colours. All figures have been reproduced using the colour-blind friendly palette of Okabe & Ito (2008).

Okabe, M., & Ito, K. (2008). Color universal design (CUD): How to make figures and presentations that are friendly to colorblind people. https://jfly.uni-koeln.de/color/

Axis labels and legends have also been changed from wheat and clover to low- and high-quality litter for consistency with descriptions in the manuscript text.

Figure 2: I would make the faceted plots in (A) and (B) all one color and a different color than those in (C) and (D). Currently, it seems as if the legend applies to (A) and (B) as well as (C) and (D).

The fill colours in faceted plots A and B have been maintained for consistency between figures with respect to the colour assigned to mineral treatments. However, to improve clarity of the composite figure, colours in facets C & D have been changed.

Figure 3: I would not call these MAOM C stocks since the authors do not account for bulk density (nor should they in an incubation). Rather, I would call this MAOM C content.

Axis label has been changed to MAOM C content as suggested. Furthermore, all reference to MAOM C stocks (with reference to the incubations) in the text has been changed to MAOM C content.

Figures 4 and 5: Please add the legends with their respective sub-figures. Having triangles for the mineral and litter types makes it confusing when all of the legends are together.

Legends have been moved adjacent to their respective sub-figures in Figures 4 and 5 as suggested. Y-axis in Figures 4C and 5C have also been changed from the absolute amount of litter-derived C to % of initial litter-derived C for consistency with other figures presented in the manuscript.

Reviewer #3 (Remarks to the Author):

The manuscript by Elias et al. presents results from a laboratory incubation in which a soil was amended with different minerals and 13C labeled litter of different quality. The aim of the study was to test the prevailing concept that high-quality, in contrast to low-quality, litter fosters MAOM formation. The authors fractionated the soil into POM and MAOM, monitored heterotrophic respiration, and traced the 13C label in fractions, respired CO2, and microbial biomass. They also analyzed the microbial community via DNA extractions. The authors found more litter-derived C in MAOM in the low-quality than high-quality litter treatment, with little effects on overall C in MAOM. They explain this by litter-quality induced shifts to a microbial community with lower CUE and priming of preexisting SOM. Notably, mineralogy determined most of the variation in litter-derived C in MAOM. The study is timely, well-written, and the methodology appears to be sound. I have two major, and many moderate and minor, comments that need to be addressed.

We thank Reviewer #3 for the helpful and constructive comments regarding the manuscript.

1. **One of the main conclusions is that mineralogy more strongly governed MAOM formation than litter chemistry. This may have simply been due to the addition of clean mineral**

surfaces without any SOM attached. This is hardly (if at all) encountered under natural conditions. So mineralogy may have a substantially weaker effect in soils in which part or all of the mineral surfaces present are covered with SOM. This limitation hampers the transferability of the study's results to natural systems and needs to be discussed to put the results into context.

It is true that clean mineral surfaces are not often encountered in natural systems and other studies exploring effects of mineralogy on SOC accumulation have used gradients of natural soils either under field or laboratory conditions. However, the use of natural soils has the disadvantage of introducing covariates to an experiment such as climate, land use and other soil properties, which must then be accounted for in subsequent analyses. The purpose of this experiment was to test specific mechanisms under highly controlled conditions, which may explain the reported inconsistent effects of litter quality on SOM formation. Whilst others have used pristine minerals to construct artificial soils for similar controlled laboratory experiments (e.g. Liang et al, 2023), here a standard soil was amended with pristine minerals to ensure microbial communities were representative of the true complexity found in soils. A study by Pronk et al, (2012) using artificial soils showed that occlusion of a significant proportion of pristine mineral surfaces (23-46%) can take place within a few days in the presence of organic matter. As pristine minerals were added to a natural soil, organo-mineral associations may have formed rapidly with pre-existing SOM prior to the start of the experiment. These organo-minerals may more closely resemble mineral particles found in natural soils.

We have added additional text to the discussion highlighting the limitations of our study in the context of pristine mineral addition to soils.

"We acknowledge that this result may be influenced by applying pristine minerals to soils, as most mineral surfaces in natural systems are already associated with organic molecules. However, a study by Pronk et al, (2012) using artificial soils found substantial sorption of OM to fresh mineral surfaces within a few days. As pristine minerals were added to a natural soil, organo-mineral associations may have formed rapidly with pre-existing SOM prior to the start of the experiment. These organo-minerals may more closely resemble mineral particles found in real soils. Nonetheless, caution should be exercised when translating these findings to natural systems." ***L347-353***

Liang, G., Stark, J. & Waring, B. G. Mineral reactivity determines root effects on soil organic carbon. Nature Communications 14, 4962 (2023). https://doi.org/10.1038/s41467-023-40768-y

Pronk, G. J., Heister, K., Ding, G.-C., Smalla, K. & Kögel-Knabner, I. Development of biogeochemical interfaces in an artificial soil incubation experiment; aggregation and formation of organo-mineral associations. Geoderma 189-190, 585-594 (2012). https://doi.org/https://doi.org/10.1016/j.geoderma.2012.05.020

2. POM was isolated by the authors, but effects of litter chemistry or mineralogy on this fraction are not discussed, although it can be important to C sequestration as well.

The focus of this study was on MAOM as it tends to persist in soils for longer than POM and is less vulnerable to environmental changes such as land management (Poeplau & Don, 2013) and climate (Benbi et al, 2014). However, we fully agree that POM can also be important for soil C sequestration, particularly in high litter producing environments such as forests or environments where mineral pools are C saturated and can persist over the longer-term in soil aggregates. Two

sentences have been added to the introduction highlighting that POM can also contribute to persistent soil C and that litter quality also controls the formation of POM.

"…However, POM can also contribute to persistent soil C through occlusion of litter fragments within soil" ***L68-69***

"Litter chemistry, in particular the C:N ratio is a primary control on litter decomposition rates, with low-quality litter (defined as litter with low mineralization rates) promoting POM formation and was previously thought to be an important control on MAOM formation by selective preservation of recalcitrant organic compounds." ***L71-74***

We found that litter quality was the predominant control on POM with low-quality litter forming more POM relative to high-quality litter. Text has been added to the discussion highlighting the differences in POM production between litters.

"Low quality litter environments typically form more POM due to slower decomposition rates promoting physical transfer of plant material through the soil profile. In agreement, we found that litter quality controlled 58.1 % of the variation in litter-C recovered as POM with low-quality litter forming more POM relative to high-quality litter." **L278-281**

Benbi, D. K., Boparai, A. K. & Brar, K. Decomposition of particulate organic matter is more sensitive to temperature than the mineral associated organic matter. Soil Biology and Biochemistry 70, 183-192 (2014). https://doi.org/https://doi.org/10.1016/j.soilbio.2013.12.032

Poeplau, C. & Don, A. Sensitivity of soil organic carbon stocks and fractions to different land-use changes across Europe. Geoderma 192, 189-201 (2013). https://doi.org/10.1016/j.geoderma.2012.08.003

More comments below:

The abstract would benefit from the placement of commas.

Thank you for the recommendation. Commas have been added and the abstract has been revised to fit within the word limit. **L23-41**

L25 Whether MAOM is dominated by microbial compounds is highly context-specific, so I suggest rephrasing this sentence.

The abstract has been revised to fit within the word limit but the remaining reference to microbial residue formation has been amended accordingly to be less definitive. *"Due to more efficient microbial anabolism, high-quality litter may produce more microbial residues for stabilisation on minerals."* **L28-30**

L61ff The authors could refer to studies that show that MAOM can also cycle on much shorter timescales than indicated in these lines.

Text has been added to the introduction highlighting that MAOM can also be cycled on shorter timescales.

"However, not all MAOM persists in the soil over long timescales as root exudates can mobilize MAOM and changes to the soil environment (e.g. redox, pH) can promote desorption by changing the solubility and surface charge of minerals. ***L63-66***

L70ff This statement contradicts those in the abstract and below, i.e., that high-quality litters are considered to boost MAOM formation (sensu Cotrufo et al., 2013).

This sentence has been revised for clarity.

"However, high-quality litter (defined as litter with high mineralization rates) is now thought to promote MAOM formation and persistence, by maximising the synthesis of microbial products that form a substantial proportion of MAOM" ***L74-77***

L86ff What about effects of litter quality on POM, which can be a substantial store of C as well?

This section of the introduction has been revised to address comments made by Reviewer #1. However, we have added two sentences at **L68-69** and **L71-74** to highlight that litter quality can influence the formation of POM as described above in response to point 2.

L97ff Occlusion and aggregation rather influence the stability of POM, if not considering particles <50 µm. The sentence is further written confusingly as it indicates that adsorption, occlusion, and aggregation form MAOM, which is probably not the meaning the authors want to convey. I suggest rephrasing the sentence for clarity.

The reference to occlusion and aggregation has been removed and the sentence rephrased.

"Variation in mineral specific surface area (SSA) and surface charge influences adsorption of organic molecules, which limits microbial access by reducing dissolved organic C concentrations in soils….." ***L119-121***

L114ff Did the authors hypothesize that mineralogy is more important than litter quality? This does not become clear here.

Yes. H2 has been rephrased for clarity *"soil mineralogy will be a more important control on the formation of MAOM relative to litter quality".* ***L147-148***

L119ff This comes surprising. I suggest incorporating information on how microbial community composition can affect SOC stability and, perhaps, also include a hypothesis on this.

The introduction has been reframed following the suggestion of Reviewer #1 to focus on the mechanisms that may explain reported inconsistent effects of litter quality on SOM formation. As part of this reframing the links between microbial physiology, community composition and SOM have been elaborated upon as quoted below. However, we do not believe a specific hypothesis is necessary here.

"Litter-microbial interactions are one mechanism proposed to explain why the effect of litter quality on SOM is inconsistent. For example, although low-quality litter may be mineralized with low microbial CUE by individual taxa, adaptation at the community level to overcome nutrient limitation may allow for decomposition without a reduction in community-level CUE." **L102-105**

L121ff The magnitude of the difference in MAOM formation between treatments amended with high- and low-quality litters could become clearer in this passage, as both litters appeared to have detrimental or no effects on overall MAOM formation.

Litter addition promoted MAOM formation relative to control treatments with no litter added. However, there was no effect of litter quality on **total** MAOM-C content. Clarification has been added.

"Here we show that soil mineralogy does act as the principal control on MAOM formation and that litter quality had no effect on total MAOM-C content". **L155-156**

L123ff This sentence is very unspecific. How did litter quality shift microbial communities?

More detail has been added describing the soil microbial community shifts in response to litter addition.

"This was associated with shifts in bacterial and fungal community composition, higher 16S ribosomal RNA copy number (a bacterial trait related to growth rate and efficiency), abundance of generalist soil saprotrophs and lower microbial CUE at early-stage decomposition." ***L157-160***

L125ff Did the authors measure the related data or is this statement purely speculative?

Yes – related data was measured. Microbial CUE was determined using ^{13}C isotope tracing at early-stage decomposition and the 16S rRNA copy number was inferred from sequencing data at both early and late-stage decomposition and used as a proxy for maximum potential growth rate and efficiency. This is presented in Table 2 and Figure 4B-D. The ability to grow rapidly in response to favourable conditions is a classic trait of copiotrophic taxa and this was associated with the formation efficiency of MAOM.

L135ff Did the authors measure the SSA of the final soil-mineral mixture? This would somehow be more informative than the SSA of the individual minerals since the SSA of the soil-mineral mixture eventually determines reactivity.

We have measured the SSA of the unamended soil and the final soil-mineral mixtures; however, we note that samples had been stored frozen, then thawed and dried for analysis. The results were as follows: control T0 unamended soil = 3.10 ± 0.01 m2/g; G-amended soil = 4.17 ± 0.02 m2/g; K-amended soil = 4.00 ± 0.02 m2/g; M-amended soil = 3.88 ± 0.01 m2/g. We note that the measured SSA of the M-amended soil is lower than might be expected if we assume a simple mixing of 10% w/w montmorillonite and the control T0 unamended soil (5.97 ± 0.22 m2/g). This could be attributable to a combination of factors including occlusion of surfaces by sorption of OM to mineral surfaces following mineral addition or the effects of freeze-thawing and drying on a 2:1 clay vs a 1:1 clay and iron oxide. Given that we used soil-mineral mixtures in our experiments that had not been frozen, thawed, and dried, we consider the SSA of the individual minerals to be more indicative of the reactivity of the soil-mineral mixtures than the above SSA analyses.

L281ff This statement is purely speculative and not supported by the results.

The language around this statement has been softened.

*"….our findings suggest that the effect of mineralogy on SOM dynamics may not be solely abiotic, and that microbe-mineral interactions could modify the effect of litter quality on MAOM formation efficiency". **L376-378***

L308ff How was litter quality determined?

Litter quality was operationally defined in this study by the C:N ratio. This has been added to the beginning of the preparation of ^{13}C-labelled litter methods section. *"Litter quality was defined in this study by the C:N ratio, which is predictive of the mineralization rate of litter."* **L406-407**

L318ff Given the authors sampled soil from an agricultural field, were there carbonates? I was wondering because the authors refer to SOC throughout, which might be incorrect when inorganic C was present in the soil.

The agricultural soil used in this study was moderately acidic (pH 5.72 ± 0.04) with no recent history of liming. Therefore, the presence of significant carbonates in this soil was unlikely. However, we analysed the dried soils for total inorganic carbon using thermogravimetric analysis. The results were as follows: control T0 unamended soil = 0.05% TIC; G-amended soil = 0.06% TIC; K-amended soil = 0.05%; M-amended soil = 0.06%. We have added a sentence to the soil collection and analysis methods highlighting that no significant inorganic carbon was present.

"Total Inorganic Carbon was quantified using Thermogravimetric analysis (LECO 700, MI, USA). No significant inorganic carbon was present in control or mineral amended soils (0.05-0.06 %)." **L433-434**

L340ff How were minerals mixed with the soils?

Pristine minerals were mixed into subsamples of homogenised soils at 10% w/w and thoroughly hand homogenised. This is described at **L456-459**.

L371/372 Why this incubation time?

This relatively long incubation time was chosen to capture both fast-decomposing and slow-decomposing litter C pools. Our intention was to end the experiment once the rate of litter-C mineralization had appeared to stabilize. However, we note that this varied between minerals and litters. This has been clarified at **L473.**

L417 50 W is the energy output of the instrument, but this does not provide any information on which energy reached the soil. I suggest calculating the energy that was delivered to the soil-SPT mixture in J/ml, as common in related studies.

We apologise for this omission. The energy delivered to the sample was 100 J/ml at an output power of 50W. This has been clarified.

"Sonication was then performed delivering 100 J ml^{-1} at a power of 50 W" ***L517-518***

L426ff Given the method well separated fractions, the fraction remaining on the 53-µm sieve should be sand only and no POM.

Yes – The fraction remaining on the 53µm sieve was sand-sized particles (>53µm) with a density >1.8g cm^3. This is referred to as "heavy POM" for consistency with the operational definitions as defined by Lavallee et al, (2020).

Lavallee, J. M., Soong, J. L. & Cotrufo, M. F. Conceptualizing soil organic matter into particulate and mineral-associated forms to address global change in the 21st century. Global Change Biology 26, 261-273 (2020). https://doi.org/https://doi.org/10.1111/gcb.14859

Response to Reviewers

We would like to thank all reviewers for their valuable insights and helpful comments, which we have addressed in the revised version of the manuscript. Reviewer comments are highlighted in bold, and our responses are in normal text. Revised sections of text quoted in the response are indicated by italicized text in quotation marks and references to changes made to the manuscript are indicated using line numbers. Line numbers refer to the tracked changes copy of the revised manuscript.

Reviewer #1 (Remarks to the Author):

The authors did a nice job of addressing my review and I don't have any further urgent comments. I think the paper deserves to be published in Nature Comm. It makes a significant advance in our understanding about the uncertain effect of litter quality on SOM stabilization – this is a question people have been pursuing for decades.

We thank Reviewer #1 for their kind words regarding the revised manuscript and their helpful comments that have improved the manuscript.

Reviewer #3 (Remarks to the Author):

The authors mosly well responded to my comments. However, I still have concerns regarding the fractionation (see below).

Newly added text in L71-74: I am not sure whether mentioning recalcitrance in this context is helpful.

The reference to "recalcitrance" has been removed and sentence rephrased.

L71-74 *"Litter chemistry, in particular the C:N ratio, is a primary control on litter decomposition rates. Low-quality litter (defined as litter with low mineralization rates) promotes POM formation and was previously thought to control SOM persistence due to molecular characteristics that limit decomposition."*

Newly added text in L74-77: I suggest adding "can" before "form" since microbial necromass in, e.g., forests can be as low as ~10%.

We agree that the importance of microbial necromass to MAOM is ecosystem specific. Text amended as suggested.

L76-78 *"…by maximising the synthesis of microbial products and residues (often referred to as necromass) that can form a substantial proportion of MAOM."*

Newly added text in L119-121: Is not rather the molecular interaction of organic matter and minerals desicive for the availability of that organic matter rather than reduced concentrations of DOC? I suggest rephrasing the sentence.

This sentence has been rephrased for clarity.

L113-115 *"Variation in mineral specific surface area (SSA) and surface charge influences the adsorption and occlusion of organic molecules, which controls the degree of microbial access to organic matter in soils…"*

L102-105 Authors may also refer to recent studies on how microbial diversity can affect necromass formation partly independently of CUE.

A sentence has been added with reference to a recent review on microbial necromass dynamics.

L98-100 *"Microbial residue accumulation in soils may also be partly independent of CUE as production rates can be influenced by other biotic factors such as microbial community interactions (Buckeridge et al, 2022)"*

Buckeridge, K. M., Creamer, C. & Whitaker, J. Deconstructing the microbial necromass continuum to inform soil carbon sequestration. Functional Ecology 36, 1396-1410 (2022). https://doi.org/https://doi.org/10.1111/1365-2435.14014

Reply to my comment on L135ff: The info given in the reply could be added to the manuscript, for clarity.

The information on the SSA of soil-mineral mixtures has been added to the methods for clarity.

L456-464 *"We also measured the SSA of the unamended soil and the final soil-mineral mixtures; The results were as follows: control T0 unamended soil = 3.10 ± 0.01 m2/g; G-amended soil = 4.17 ± 0.02 m2/g; K-amended soil = 4.00 ± 0.02 m2/g; M-amended soil = 3.88 ± 0.01 m2/g. The measured SSA of the M-amended soil is lower than might be expected if a simple mixing of 10% w/w montmorillonite and the control T0 unamended soil (5.97 ± 0.22 m2/g) is assumed. This could reflect occlusion of surfaces by sorption of OM to mineral surfaces following mineral addition or the effects of freeze-thawing and drying on a 2:1 clay vs a 1:1 clay and iron oxide as soils were stored frozen, then thawed and dried for analysis. We therefore consider the SSA of the individual minerals to be more indicative of the reactivity of the soil-mineral mixtures than the above SSA analyses."*

L417 sonication energy: The delivered energy appears to be very low (see e.g., Just et al. https://doi.org/10.3389/fsoil.2021.692583). It is thus questionable whether the authors properly dispersed the soil. Why was this low energy output chosen? This does not hamper comparability among samples in the current study, but it is questionable whether the results can be compared with those of other studies or transferred to properly dispersed soils. I acknowledge that sandy loam may require lower energies than clay-rich soils; however, even sandy loam can contain ~20% of clay, for which the chosen energy appears to be low.

Whilst a higher sonication energy of ~450 J ml^{-1} has been shown to be suitable for complete aggregate dispersion across a wide range of soils (Schrumpf et al, 2013, Schmidt et al, 1999), this energy may also cause the redistribution of organic carbon across fractions by disrupting weak organic-sand associations, comminuting particulate organic matter that may increase retention in the heavy mineral fraction (Buks et al, 2021) and increasing mineral contamination of light fractions (Cerli et al, 2012). As suggested by our reviewer and others this may be most pertinent for sandy soil such as that used in this experiment (Sandy loam: 9 % clay, 24 % silt, 67 % sand). As part of our protocol, we initially identified the minimum sonication energy that was required for aggregate disruption in our soils (following removal of the free light fraction) by observing the effects of a stepwise increase of sonication energy on liberated light fraction (LF) material. Sonication energy was applied in 50 J ml^{-1}

increments from 50-300 J ml^{-1} and LF material removed by pipetting prior to each subsequent sonication of 50 J ml^{-1}. Complete disruption of aggregates was assumed when no further LF material was observed floating in the SPT solution. Soils were weakly aggregated as 100 J ml^{-1} was sufficient to fully disperse our soils as no further LF material was released after subsequent sonication steps. Although as highlighted, this is at the low end of sonication energies reported in other SOM fractionation procedures, it is in line with sonication energies reported for aggregate disruption in coarse textured soils (Schrumpf et al, 2013). Although Just et al, (2021) do report that with increasing dispersion intensity (100-450 J ml^{-1}), OC content tended to slightly decrease in >20μm and increase in <20μm fractions, the effect was most pronounced in fine textured soils. The quality indicators reported (indicating the isolation efficiency of two SOC fractions with distinct turnover rates) also only differed between 100 J ml^{-1} and 450 J ml^{-1} ultrasonication energies in the most clay-rich soil whilst for soils with lower clay contents there was no difference in the isolation quality. It is also possible that soils amended with pristine minerals behave differently under sonication in contrast to natural soils. Given that we tested the dispersion energy required for our soils, we believe that our results can be compared to other studies. This justification for the sonication energy used has been added to the methods.

L521-528 *"To minimise the redistribution of C across fractions (Cerli et al, 2012), the lowest energy required to fully disperse soils was pre-determined by observing the effects of a stepwise increase of sonication energy on liberated light material. Sonication energy was applied in 50 J ml^{-1} increments up to 300 J ml^{-1} and floating light material removed by pipetting prior to each subsequent sonication of 50 J ml^{-1}. Complete disruption of aggregates was assumed when no further material was observed floating in the SPT solution. Soils were weakly aggregated as 100 J ml^{-1} was sufficient to fully disperse our soils. Although this sonication energy is low, others have found 100 J ml^{-1} to be sufficient for the dispersal of coarse textured soils (Schrumpf et al, 2013, Just et al, 2021)."*

Schrumpf, M. et al. Storage and stability of organic carbon in soils as related to depth, occlusion within aggregates, and attachment to minerals. Biogeosciences 10, 1675-1691 (2013). https://doi.org/10.5194/bg-10-1675-2013

Schmidt, M. W. I., Rumpel, C. & Kögel-Knabner, I. Evaluation of an ultrasonic dispersion procedure to isolate primary organomineral complexes from soils. European Journal of Soil Science 50, 87-94 (1999). https://doi.org/https://doi.org/10.1046/j.1365-2389.1999.00211.x

Cerli, C., Celi, L., Kalbitz, K., Guggenberger, G. & Kaiser, K. Separation of light and heavy organic matter fractions in soil — Testing for proper density cut-off and dispersion level. Geoderma 170, 403-416 (2012).
https://doi.org/https://doi.org/10.1016/j.geoderma.2011.10.009

Büks, F., Kayser, G., Zieger, A., Lang, F. & Kaupenjohann, M. Particles under stress: ultrasonication causes size and recovery rate artifacts with soil-derived POM but not with microplastics. Biogeosciences 18, 159-167 (2021). https://doi.org/10.5194/bg-18-159-2021

Reply to my comment on L426ff: The authors separated fPOM and oPOM via density using SPT. As such, the fraction on top of the 53-μm sieve after separtion of POM should be sand only without any POM at all, given that the authors' separation was effective. It does not make sense to then again combine the presumably clean POM fractions the authors isolated via density with the mineral sand fraction on the 53-μm sieve (as indicated in L527), as use of SPT would not have been needed in the first

place. The description in the methods is either confusing or the authors have not understood the rationale for separating POM via SPT.

Thank you for highlighting this point. We summed the litter C recovered as light POM with that recovered in the sand-sized (>53μm) mineral material (previously referred to as heavy POM) (as presented in Figure 2) as in dispersed soils C recovered in this sand-sized fraction can represent organic debris encrusted in fine minerals that thus remains in the heavy fraction during density separation. However, C in the sand-sized pool can also be stabilised through association between small organic molecules and sand-sized particles. Given that we cannot discriminate between these two stabilisation mechanisms and, as pointed out the sum of light POM and sand-sized mineral material is analogous to the composite POM fraction isolated through physical size fractionation (53μm) alone, we acknowledge that they should be presented and discussed independently. 0.2-6.9 % of litter-C was recovered as POM and 0.6-6.4 % was associated with the sand-sized fraction. Across all mineral treatments, more C from low-quality litter was recovered in both POM and the sand-sized fraction than from high-quality litter. Figure 2c and associated statistics have been updated to include both POM and sand-sized SOM. Discussion of the treatment effects on POM and sand-sized SOM have been added to the results and to the discussion as quoted below:

L186-194 *"More litter-C was also stabilised as sand-sized SOM (> 1.8 g cm^{-3}, >53μm; sometimes called heavy POM) from low- relative to high-quality litter across all soil treatments (Figure 2c) although the proportion of added litter-C recovered in this pool was low (0.6-6.4 %). In contrast to MAOM, litter quality explained most variation in litter-C recovered as sand-sized SOM (49.8 %) (Table S2). 0.2-6.9 % of added litter-C was recovered as light POM (Figure 2b-c). Litter quality explained 33.5 % of the variation in litter-C recovered as POM while soil mineralogy explained 49.7 % (Table S2). Across all mineral treatments, more C from low-quality litter was recovered as light POM than from high-quality litter (Figure 2b-c)."*

L275-281 *"Low-quality litter environments typically form more POM due to slower decomposition rates, promoting physical transfer of plant material through the soil profile, whilst sand-sized SOM appears sensitive to the quality of organic C inputs. In agreement, we found that more litter-C was recovered as both POM and sand-sized SOM from soils amended with low- relative to high-quality litter. However, the proportion of litter-C recovered in these pools was low relative to MAOM."*

Reviewer #4 (Remarks to the Author):

Overall comments: I appreciate the authors attention to my comments and those of the other reviewers. I am mostly satisfied with the resubmitted manuscript and am excited for it to contribute to the literature. The only bigger concern I have with the resubmitted manuscript is the very low CUE reported in this experiment (largely below 0.1). While this CUE isn't outside of the realm of possibility, these low values certainly deserve some speculation about why they are so low and some defense from the authors.

We thank Reviewer #4 for their positive comments. The low CUE values reported in the manuscript are most likely a function of the litter substrate used to quantify ^{13}C incorporation into microbial biomass and the duration of the incubation. These two factors vary widely across studies that measure soil microbial CUE and make comparison between studies challenging. We chose to conduct a longer incubation than is standard using e.g. ^{13}C glucose as a substrate because we applied litter as particulate material and wanted to allow

time for microbial assimilation and to integrate the effect of shifts in microbial community composition in response to litter inputs. However, we acknowledge that the longer incubation duration may also include some effect of microbial turnover and substrate recycling. For this reason, we do not attempt to make any comparisons of other published CUE values and limit our interpretation to treatment comparisons within our study and the implications. This justification and caveats have been added to the manuscript discussion.

L298-304 *"We caution that the use of ^{13}C litter tracing to calculate CUE will likely reflect a subset of the microbial community active on the litter substrate and declines with incubation duration due primarily to increased cumulative respiration of ^{13}C. We chose a longer incubation duration (15 days) to integrate the effects of shifts in microbial community composition in response to litter inputs resulting in low CUE values. However, we acknowledge that this long incubation potentially also includes the effect of turnover and substrate recycling and make no comparison to other published CUE estimates".*

Other minor comments:

Line 67: POM turns over faster on average – see Heckman, Hicks-Pries et al., 2022 for overlapping turnover times of POM and MAOM.

Heckman, K., Hicks Pries, C.E., Lawrence, C.R., Rasmussen, C., Crow, S.E., Hoyt, A.M., von Fromm, S.F., Shi, Z., Stoner, S., McGrath, C., Beem-Miller, J., Berhe, A.A., Blankinship, J.C., Keiluweit, M., Marin-Spiotta, E., Monroe, J.G., Plante, A., Schimel, J., Sierra, C.A., Thompson, A., Wagai, R., 2022. Beyond bulk: Density fractions explain heterogeneity in global soil carbon abundance and persistence. Global change biology 28, 1178-1196.

The language has been softened and further detail added to highlight that whilst in general MAOM does turnover more slowly, this varies across ecosystems and is determined by whether POM is free or occluded.

L65-68 *"Particulate organic matter (POM) comprises primarily lightweight fragments of plant material that generally turnover faster than MAOM although this varies across ecosystems (Heckman et al, 2022). POM can also turnover more slowly and contribute to persistent soil C through occlusion of litter fragments within soil aggregates"*

Lines 78-94: The empirical evidence for MEMS and for the Castellano et al., 2015 paper are reviewed in Rocci et al., 2024 if the authors want a more recent review of the evidence for these therories.

Rocci, K., Cotrufo, M.F., Ernakovitch, J., Foster, E., Frey, S., Georgiou, K., Malhotra, A., Reich, P., Schlerman, E., Wieder, W.R., 2024. Bridging 20 years of soil organic matter frameworks: empirical support, model representation, and next steps. Journal of Geophysical Research Biogeosciences.

Reference to Rocci et al, 2024 added at **L89**.

Line 93: I feel the mention of SOC models here is too brief to be usedul and I suggest remocing it. Particularly, it is unclear what "current generation" models are? Are these microbially implicit models?

Reference to SOC models has been removed and sentence rephrased.

L91-94 *"To resolve uncertainties regarding the relationship between litter quality and SOM persistence requires greater consideration of the interactions between litter quality, microbes, the mineral matrix, and the abiotic environment."*

Lines 109-111: I would also cite more fundamental nutrient mining papers here such as Moorhead and Sinsabaugh, 2006 and Chen et al., 2014, where Chen et al., 2014 also found that oligotrophs drive the priming effect.

Chen, R., 2014. Soil C and N availability determine the priming effect: microbial N mining and stoichiometric decomposition theories. Global Change Biology 20, 2356–2367, doi: 10.1111/gcb.12475

Moorhead, D.L., Sinsabaugh, R.L., 2006. A theoretical model of litter decay and microbial interaction. Ecological Monographs 76, 151-174.

Additional citations to Moorhead & Sinsabaugh, (2006) and Chen et al, (2014) added at **L106**

Lines 255-257: Is this line different from the preceding one because the specialist saprotrophs are not influenced by litter quality?

This section was unclear and has been revised for clarity as quoted below

L240-250 *"At early-stage decomposition the relative abundance of generalist soil saprotrophs and specialist litter saprotrophs were both strongly influenced by litter quality (Table S7, Figure S2b). Within each soil, the relative abundance of generalist soil saprotrophs was positively correlated with litter-C respired, driven by higher abundance in high- relative to low-quality litter treatments (Figure S2c). Conversely, the relative abundance of specialist litter saprotrophs was negatively correlated with litter-C respired due to higher abundance in low- relative to high-quality litter treatments (Figure S2d). Soil mineralogy also controlled the relative abundance of specialist litter saprotrophs with lower abundance in montmorillonite soils (Table S7, Figure S2b). Across soils (within each litter addition treatment), specialist litter saprotroph abundance was positively associated with the amount of litter-C respired (Figure S3)."*

Response to Reviewers

We would like to thank Reviewer #3 for their helpful comments, which we have addressed as detailed below. Reviewer comments are highlighted in bold, and our responses are in normal text.

REVIEWERS' COMMENTS

Reviewer #3 (Remarks to the Author):

The authors have tackled my comments well. However, I am still not convinced of "sand-sized SOM."

I am not aware of any study that has shown that the "sand-sized fraction can represent organic debris encrusted in fine minerals," and without appropriate citations or experimental evidence, this statement is not credible, as the C in the sand fraction can simply be an artifact of the fractionation procedure.

I thus suggest either providing references/experimental evidence for the statement above or not discussing sand-sized SOM in the manuscript.

All reference to sand-sized SOM has been removed from the manuscript results and discussion to focus on the two primary fractions; MAOM and POM. The fractionation methods now describe that the sand fraction (>53μm) was also isolated by the fractionation procedure but is not discussed.

"All dried fractions were ground to a fine powder using a Retsch mixer mill (MM400, Retsch, Dusseldorf, Germany) and analysed for C and δ13C as described above. Sand particles (>53 μm) retained on the sieve (sometimes termed sand-sized SOM or heavy POM) were also dried, weighed and analysed for C and δ13C but are not discussed." **L520-523**
